# Prevalence of menthol cigarette use among adults who smoke from the United States by census division and demographic subgroup, 2002–2020: findings from the International Tobacco Control (ITC) project

**DOI:** 10.1186/s12963-024-00326-0

**Published:** 2024-04-09

**Authors:** Pete Driezen, Shannon Gravely, Karin A. Kasza, Mary E. Thompson, K. Michael Cummings, Andrew Hyland, Geoffrey T. Fong

**Affiliations:** 1 Department of Psychology, University of Waterloo, Waterloo, ON Canada; 2 School of Public Health Sciences, University of Waterloo, Waterloo, ON Canada; 3 Department of Health Behavior, Roswell Park Comprehensive Cancer Center, Buffalo, NY USA; 4 Department of Statistics and Actuarial Science, University of Waterloo, Waterloo, ON Canada; 5 Psychiatry and Behavioral Sciences, Medical University of South Carolina, Charleston, SC USA; 6 Ontario Institute for Cancer Research, Toronto, ON Canada

**Keywords:** Menthol cigarettes, Prevalence, United States, Multi-level regression and post-stratification, Sociodemographics

## Abstract

**Background:**

Targeted marketing of menthol cigarettes in the US influences disparities in the prevalence of menthol smoking. There has been no analysis of sub-national data documenting differences in use across demographic subgroups. This study estimated trends in the prevalence of menthol use among adults who smoke in the nine US census divisions by sex, age, and race/ethnicity from 2002 to 2020.

**Methods:**

Data from 12 waves of the US ITC Survey were used to estimate the prevalence of menthol cigarette use across census divisions and demographic subgroups using multilevel regression and post-stratification (*n* = 12,020). Multilevel logistic regression was used to predict the prevalence of menthol cigarette use in 72 cross-classified groups of adults who smoke defined by sex, age, race/ethnicity, and socioeconomic status; division-level effects were fit with a random intercept. Predicted prevalence was weighted by the total number of adults who smoke in each cross-classified group and aggregated to divisions within demographic subgroup. Estimates were validated against the Tobacco Use Supplement to the Current Population Survey (TUS-CPS).

**Results:**

Overall modeled prevalence of menthol cigarette use was similar to TUS-CPS estimates. Prevalence among adults who smoke increased in each division from 2002 to 2020. By 2020, prevalence was highest in the Middle (46.3%) and South Atlantic (42.7%) and lowest in the Pacific (25.9%) and Mountain (24.2%) divisions. Prevalence was higher among adults aged 18–29 (vs. 50+) and females (vs. males). Prevalence among non-Hispanic Black people exceeded 80% in the Middle Atlantic, East North Central, West North Central, and South Atlantic in all years and varied most among Hispanic people in 2020 (Pacific: 26.5%, New England: 55.1%).

**Conclusions:**

Significant geographic variation in the prevalence of menthol cigarette use among adults who smoke suggests the proposed US Food and Drug Administration (FDA) menthol cigarette ban will exert differential public health benefits and challenges across geographic and demographic subgroups.

**Supplementary Information:**

The online version contains supplementary material available at 10.1186/s12963-024-00326-0.

## Introduction

The targeted marketing of menthol cigarettes in the United States (US) has influenced disparities in the prevalence of menthol cigarette use across demographic groups. Targeted marketing is a common practice used by cigarette manufacturers to increase sales in specific demographic groups [1, 2]. Beginning in the 1950s and 1960s, menthol cigarette brands gained popularity in the US over concerns about the health risks of smoking [2]. At that time, cigarette companies developed regionally focused campaigns to increase sales, capitalizing on regional demographic differences to explicitly market menthol cigarette brands to Black and Hispanic people [1–4], as well as to women and youth [2].

Recent estimates from the Tobacco Use Supplement to the Current Population Survey (TUS-CPS) and the National Survey on Drug Use and Health (NSDUH) indicate that, in 2019, 79–85% of Black people who smoke reported currently smoking menthol cigarettes compared to only 25–40% of White people who smoke [5, 6]. Menthol cigarette use has also become more popular among Hispanic people who smoke. By 2019, 36–48% of Hispanic adults who smoke reported smoking menthol cigarettes [5, 6]. In April 2022, the US Food and Drug Administration (FDA) announced a product standard that would prohibit menthol as a characterizing flavor in cigarettes [7], with the expectation that it will reduce tobacco-related disparities in communities where menthol cigarette smoking has been more prevalent [5].

Understanding how disparities in menthol use have evolved over time can inform evaluations of the equity impact of tobacco control policies at the population level [8]. Ongoing surveillance is needed to assess whether tobacco-related disparities are influenced by changes in policies intended to reduce those disparities [8]. National estimates from TUS-CPS and NSDUH demonstrate the prevalence of menthol use among adults who smoke increased since 2003 among most demographic groups [5, 6]. These increases correspond to increases in the market share of menthol cigarettes during this time [9, 10]. While broad regional differences in menthol use exist in the US [5], few studies have examined sub-national trends in the prevalence of menthol use among adults who smoke below the level of census regions. The purpose of this study was to estimate long-term trends in the prevalence of menthol cigarette use among adults who smoke in the US across the nine census divisions from 2002 to 2020. By estimating division-specific trends in menthol use among specific demographic groups, it is possible to examine whether disparities in menthol use changed within US census divisions since 2002.

## Methods

### Multilevel regression and post-stratification

Multilevel regression and post-stratification (MrP) is a statistical small area estimation method that uses population survey data to estimate reliable statistics for subgroups for which the survey was not designed. These subgroups, or “domains”, are usually comprised of small sample sizes and are often, but not necessarily, defined by geographic boundaries. First proposed by Gelman and Little [11], MrP relies on two steps. The first step fits a multilevel (or mixed effects) regression model to predict an outcome for different groups of respondents based on their sociodemographic characteristics [11, 12]. The second step uses post-stratification methods to average estimates across categories proportionally to the size of each group in the population [11, 12]. The advantage of this approach is that it uses many groups of respondents to produce weighted average estimates for small domains of interest. In this way, the method borrows strength from similar groups of respondents across domains [11].

In the first step, a multilevel regression model is fit using respondent-level information, i.e., the outcome of interest and relevant sociodemographic predictors. The model utilizes a multilevel data structure, such that respondents are nested within domains. These domains are fit as random intercepts in the model. Predictions are made from the fitted model for the outcome, such as the proportion of adults using menthol cigarettes among all adults who currently smoke.

The post-stratification step requires a disaggregated data frame describing the joint distribution of the number of people within each cell for which predictions are made in a given domain; for example, the number of males aged 18–29 having a high school education who currently smoke, the number of females aged 18–29 having a high school education who currently smoke, etc., for all cross-classified demographic groups. The post-stratification step then uses model predictions to average category-specific estimates to the domain level in proportion to their population size [11, 12], yielding an overall weighted average for each domain.

### Data sources

#### The International Tobacco Control US Surveys

The International Tobacco Control (ITC) US Surveys are part of a larger set of multi-country surveys: the ITC Four Country Survey (ITC 4 C) and the ITC Four Country Smoking and Vaping Survey (ITC 4CV). These projects were designed to evaluate the effects of national-level tobacco control policies [13, 14]. The original ITC 4 C Survey was a prospective cohort survey of nationally representative samples of adults (ages 18 and older) who smoked at least monthly in Canada, the US, the United Kingdom, and Australia. For the US arm of the study, a stratified sampling design was used to recruit respondents into the survey via random-digit dialing [14]. In subsequent survey years, respondents lost to attrition were replaced with newly recruited respondents using the same sampling methods. Data were collected via computer-assisted telephone interviewing (CATI); beginning in 2008 (Wave 7), respondents had the option of completing the survey online. The increasing costs of telephone surveys and recruiting respondents by telephone led to a shift in recruitment methods in 2015 (second half of Wave 9 in the US). At that time, the GfK commercial panel (later Ipsos) was used to recruit new respondents into the survey. A new iteration of ITC 4 C began in 2016. In Wave 1 of ITC 4CV, respondents from the original ITC 4 C survey who completed Wave 9 were invited to participate in the new survey. New respondents for this survey were also recruited from GfK. All data were collected via web surveys in the new study. Both cross-sectional and longitudinal (except Wave 1) survey weights were computed for all survey waves, using post-stratification and calibration for ITC 4 C and raking (or iterative proportional fitting) methods for ITC 4CV. Supplementary Table 1 (see Additional file 1) summarizes the key features of both surveys, including the sampling design, survey weights, data collection time frame, data collection mode, and wave-specific sample sizes of adult respondents who reported smoking at least monthly. All respondents participating in the ITC 4 C and ITC 4CV surveys provided informed consent prior to completing a survey.

#### The US Behavioral Risk Factor Surveillance System

Data from the US Behavioral Risk Factor Surveillance System (BRFSS) and the American Community Survey (described below) were used to construct the post-stratification data frame for MrP modeling. Since its inception, BRFSS has been implemented as a state-level survey, randomly sampling adult respondents (aged 18 and older) within all 50 states, the District of Columbia, and three territories using a stratified design [15]. Initially, state-level BRFSS surveys used RDD to sample respondents [16]. As a result of increasing cell phone ownership, in 2011, sampling was conducted using an overlapping landline and cell phone sampling frame [17, 18]. In that same year, BRFSS revised its weighting methodology, moving from post-stratification to raking methods [15, 18]. Supplementary Table 1 (Additional file 1) summarizes the BRFSS while a complete description of its underlying methodology is available elsewhere [16–19]. Public use data files used in this study were obtained from the CDC [20].

#### The American Community Survey

The American Community Survey (ACS) is the largest household survey conducted in the US; it was designed to provide detailed information on the demographic characteristics of the population for small geographic units [21, 22]. Complete methodological details are described elsewhere [23]. This study used data provided in the one-year public use microdata samples (PUMS), available from the US Census Bureau [24].

#### The Tobacco Use Supplement to the Current Population Survey

Data from the Tobacco Use Supplement to the Current Population Survey (TUS-CPS) were used to validate division-level modeled estimates of the prevalence of menthol cigarette use among US adults who smoke. The TUS-CPS is the largest nationally representative survey of adult tobacco use in the US and derives its sample from respondents aged 18 and older who completed a CPS interview [25, 26]. Administered by the US Census Bureau and the Bureau of Labor Statistics, the CPS is a monthly household survey of the non-institutionalized population aged 16 and older, providing monthly employment and demographic statistics for the US workforce [26]. CPS uses a two-stage stratified cluster design to randomly select 60,000 household units each month for interview [26]. The Tobacco Use Supplement to the CPS has been conducted every three to four years since 1992 [27]. The current study used publicly available data from TUS-CPS harmonized data file containing data from all waves of the TUS-CPS [28].

### Measures

#### Outcome measure: Menthol cigarette use

 In Waves 1 through 4 of the ITC 4 C Surveys, respondents who smoked were asked to report whether their current brand of cigarettes or the brand they last purchased was menthol, plain, or some other flavor. All respondents who reported their brand was menthol were classified as smoking menthol (vs. not smoking menthol). In the remaining waves of ITC 4 C and the first three waves of ITC 4CV, respondents who smoked at least monthly were asked to report the brand of cigarettes they usually smoked as well as the brand of cigarettes they last purchased. Respondents reporting brands containing the term “menthol” to either regular or last purchased brand were classified as smoking menthol. Beginning in Wave 7 of ITC 4 C, respondents who reported smoking “Camel Crush” cigarettes (regular or last purchased brand) were also classified as smoking menthol.

Beginning in 2003 (Wave 6), a question about current use of menthol cigarettes was added to the TUS-CPS. In that wave and all subsequent waves, respondents were asked “Do you usually smoke menthol or non-menthol cigarettes?” Those respondents who reported “’menthol” were classified as smoking menthol while respondents who reported “non-menthol” or “no usual type” were classified as not smoking menthol.

#### Covariates

Sociodemographic measures from the ITC 4 C and 4CV Surveys used as covariates for fitting the multilevel models were defined in the same way (or as closely as possible for household income) as measures from BRFSS and ACS, the data sources used to construct the post-stratification frame. Census division was defined using respondents’ state of residence: New England (CT, ME, MA, NH, RI, VT), Middle Atlantic (NJ, NY, PA), East North Central (IN, IL, MI, OH, WI), West North Central (IA, KS, MN, MO, NE, ND, SD), South Atlantic (DC, DE, FL, GA, MD, NC, SC, VA, WV), East South Central (AL, KY, MS, TN), West South Central (AR, LA, OK, TX), Mountain (AZ, CO, ID, MT, NM, NV, UT, WY), and Pacific (AK, CA, HI, OR, WA). Other measures were sex (female, male), age group (18–29, 30–49, $\ge$ 50), and race/ethnicity (exclusively non-Hispanic White, exclusively non-Hispanic Black, Hispanic (with or without reporting other racial groups), and all other non-Hispanic groups).

Socioeconomic status (SES) was defined as a composite measure that combined highest level of education and annual household income. To construct this measure, education was classified as more than a high school education vs. otherwise (including education not reported). In the ITC data, income was classified as earning $45,000/year or more vs. otherwise (including income not reported). In the BRFSS and ACS data, due to the way income was provided on the BRFSS public use data files, income was classified as earning $50,000/year or more vs. otherwise (including income not reported). A three-category measure of SES, adapted from Kasza et al. [29] and Licht et al. [30], was defined on the basis of these two binary measures. Respondents having more than a high school education *and* household incomes $$$$$$$$$$\geq$$$45,000/year were classified as having a “high” SES. Respondents having a high school education or less (including not reported) *and* reported earning $$$$$$$$$$<$$$45,000/year (or not reporting income) were classified as having a “low” SES. All other respondents were classified as having a “moderate” SES.    

The multilevel regression models also included a division-level fixed effect for the labor force participation rate to account for underlying economic trends during the time period modeled (2002 to 2020). Division- and year-specific estimates of the labor force participation rate were obtained from the US Bureau of Labor Statistics [31]. Estimates across all survey years were mean centred within census divisions. Temporal trends, described in the next section, were modeled using fixed effects for survey wave.

### Statistical analysis

#### MrP step 1: Multilevel logistic regression

Nine different multilevel logistic regression models were fit to predict the prevalence of menthol cigarette use among adults who currently smoke in each census division for all ITC survey years. Given the extended data collection period for Wave 9 of ITC 4 C, the data for this wave were split into two sub-waves: (a) 2013–2014 and (b) 2015, yielding 13 “waves” of data to model trends in the prevalence of menthol use from 2002 to 2020. All sociodemographic measures (sex, age group, race/ethnicity, and SES) were fit as main effects. In all models, two-way interactions were fit (sex-by-age group, sex-by-race/ethnicity, and age group-by-race/ethnicity) so that the three-way sex-by-age group-by-race/ethnicity interaction could be modeled. The division-level mean-centred labor force participation rate was fit as a fixed effect (“lfParticC”). Age group, SES, and the labor force participation rate were fit as time-varying covariates while all other covariates were time invariant.

Different models fit different temporal trends. In Model 1, time was fit as a linear trend where the first wave was indexed at time = 0. In Model 2, time was fit as a categorical indicator. Piecewise linear trends (PWLT) were fit in all remaining models; these models allowed for a “bend” in the fitted temporal trend. The first PWLT (“timeCC”) allowed for a bend beginning in 2008, shortly after Camel Crush cigarettes were introduced into the US market. A second PWLT modeled a lagged effect for the introduction of Camel Crush cigarettes. This trend (“lagCC”) allowed for a bend beginning in 2010, when there would have been greater market penetration of Camel Crush cigarettes. The third PWLT allowed for a bend in the temporal trend beginning in 2016, representing the first wave of the ITC 4CV survey (“time4CV”). Two additional models allowed for two bends in the temporal trend: one model fit the temporal trend as “time + timeCC + time4CV” and the second fit the trend as “time + lagCC + time4CV.”

Census divisions were fit as a random intercept in all estimated models using a variance components covariance structure. Model 8 fit race/ethnicity as an additional random intercept nested within census divisions. Model 9 also fit two random intercepts: one for census division and a second for survey wave. The Akaike Information Criterion (AIC) and Bayesian Information Criterion (BIC) were used to select the final best fitting model. With the exception of model 9, all models incorporated the cross-sectional ITC Survey weights; these weights were rescaled to sample size within census divisions for each survey wave. A division-level weight was also specified; this weight was set to a value of 1 because there was no sampling of divisions in the ITC US Surveys. All models were fit using PROC GLIMMIX in SAS (Version 9.4 TS1M7, SAS/STAT 15.2). SAS PROC PLM was used to predict the probability of menthol use ($\widehat{\pi }$) based on the multilevel model for the 72 cross-classified demographic groups of adults who currently smoke for each census division in all survey years. In other words, predictions were made for all demographic groups defined by the crossing of sex, age group, race/ethnicity, and SES in each census division for all ITC survey years, yielding 8,424 predictions (i.e., $2\text{*}3\text{*}4\text{*}3\text{*}9\text{*}13=8,424$).

#### Construction of the post-stratification frame

For each year in which an ITC Survey was conducted, data from the contemporaneous BRFSS and ACS surveys were used to estimate the total number of people who smoked in each census division for the 72 cross-classified demographic groups. These totals were estimated by combining direct, survey-based estimates of the prevalence of smoking among adults from BRFSS with model-based estimates, predicted from a logistic regression model. First, the prevalence of current smoking within census division for each cross-classified demographic group was estimated using SAS-callable SUDAAN (Version 11.0.3) to account for the sampling design employed in BRFSS and the sampling weights. A logistic regression model was then fit using SAS PROC SURVEYLOGISTIC to model the probability of current smoking using census division, sex, age group, race/ethnicity, SES, and four two-way interaction effects: division-by-ethnicity, sex-by age group, sex-by-ethnicity, and age group-by-ethnicity. For each cross-classified group (or post-stratification cell), the predicted probability of current smoking, and its associated standard error, was estimated from fitted model parameters using SAS PROC PLM.

Direct and model-based estimates were then combined to form a composite estimator of small domain smoking prevalence to balance the potential bias of the model-based estimate against the instability of the direct estimate [32]. This combined estimate gives more weight to the direct estimate if the variance of the model-based estimate is large relative to the direct estimate. The composite estimate ${\theta }_{i}$ for a given small domain was computed as

1 $$ \begin{array}{c}{\theta }_{i}=\left({a}_{i}\text{*}{p}_{id}\right)+\left(\left(1-{a}_{i}\right)\text{*}{p}_{im}\right)\end{array}$$

where, for each small domain $$ i$$:

${p}_{id} =$ the direct estimate of prevalence,

${p}_{im}=$ the model-based estimate of prevalence, and

$$ {a}_{i}= $$ the relative variance of $$ {p}_{im}$$ to the total variance, i.e., $$ {a}_{i}=\frac{{v}_{im}}{{v}_{im}+{v}_{id}}$$, where

${v}_{im}=$ the variance of the model-based estimate, and

${v}_{id}=$ the variance of the direct estimate.

Depending on the available sample size contributing to the direct estimate of smoking prevalence in each post-stratification cell, either the composite estimate of prevalence $$ {\theta }_{i}$$ was used to compute the total number of adults who smoked in that small domain or the model-based estimate $$ {p}_{im}$$ was used. The model-based estimate was used if any of the following criteria were met for a given post-stratification cell:

- the cell sample size $$ <$$ 5,
- the direct estimate of prevalence = 0,
- the direct estimate of prevalence = 1, or.
- the cell size * direct estimate < 1 (i.e., the estimated number of adults who smoked for that post-stratification cell was less than one).

The estimated smoking prevalence for a given cell was then multiplied by the total population size, estimated from the ACS, for that cell to obtain an estimate of the total number of people who smoked in that cell. These totals were adjusted so that the total number of people who smoke for each primary demographic category at the census division level matched the estimated number of people who smoked at the census division level from BRFSS. Using males as an example, the 72 post-stratification totals within a census division were adjusted so that the total number of males who smoked estimated from the post-stratification totals matched the marginal number of males who smoked from BRFSS for that census division. These adjustments were conducted using iterative proportional fitting in R (Version 4.3.1) using the “mipfp” library (Version 3.2.1) [33]. Put another way, small domain post-stratification totals for each census division were adjusted so that the sum of those totals for each overall demographic group matched the estimated number of adults who smoked in that same demographic group from the BRFSS survey.

#### MrP step 2: Post-stratification

In the post-stratification step, the estimated prevalence $\widehat{\pi }$ of menthol use from the multilevel logistic regression model was weighted using the post-stratification totals to obtain a final estimate of the prevalence of menthol use for each census division in each survey year [34, 35]. Thus, the census division- and year-specific menthol prevalence ${\widehat{p}}^{cd,t}$ is a weighted average:

2 $$ \begin{array}{c}{\widehat{p}}^{cd,t}=\frac{\sum _{j=1}^{72}\left({N}_{j}^{cd,t}\text{*}{\widehat{\pi }}_{j}^{cd,t}\right)}{\sum _{j=1}^{72}{N}_{j}^{cd,t}} \end{array}$$

where,

$$ {N}_{j}^{cd,t} = $$ the post-stratification total number of adults who smoke in demographic group $$ j$$ in census division $$ cd$$ and survey year $$ t$$,

$$ {\widehat{\pi }}_{j}^{cd,t} = $$ the predicted probability of menthol use in demographic group $$ j$$ in census division $$ cd$$ and survey year $$ t$$.

Prevalence estimates for specific population subgroups were obtained by considering only the relevant subgroup of interest (e.g., all adult females who currently smoke, all non-Hispanic Black adults who currently smoke) and aggregating to the division level for each survey year.

#### Confidence intervals

Confidence intervals were estimated using a non-parametric bootstrapping technique [36]. Briefly, SAS PROC SURVEYSELECT was used to randomly select 1000 samples of ITC respondents within census division and survey year using unrestricted random sampling (i.e., selection with equal probability and with replacement). The size of each replicate sample equaled the total number of ITC respondents who reported smoking cigarettes in a given census division and survey year. The final multilevel regression model (Table 1) was fit using each of these replicate samples and the division-level prevalence of menthol use was estimated in the post-stratification step yielding 1000 estimates of prevalence. 95% confidence interval were estimated using the 2.5th and 97.5th values [36].

**Table 1:** Model fit statistics and type 3 tests of fixed effects for nine mixed effects logistic regression models estimating the probability of smoking menthol cigarettes among US adults who currently smoke from 2002 to 2020

	Model 1	Model 2	Model 3	Model 4	Model 5	Model 6	Model 7	Model 8	Model 9
*Statistic*									
# of Model Parameters	29	40	30	30	30	31	31	32	32
Log L	23555.83	23529.13	23553.18	23553.60	23555.33	23546.93	23545.16	23486.97	24520.09
AIC	23613.83	23609.13	23613.18	23613.60	23615.33	23608.93	23607.16	23550.97	24584.09
BIC	23619.55	23617.01	23619.10	23619.52	23621.24	23615.04	23613.27	23557.28	24520.09
*Fixed effects: Type 3 F test (p-value)*									
Female	6.54 (0.011)	6.62 (0.010)	6.65 (0.010)	6.68 (0.010)	6.52 (0.011)	6.54 (0.011)	6.59 (0.010)	6.68 (0.010)	40.48 (< 0.001)
Age group	52.69 (< 0.001)	44.99 (< 0.001)	46.72 (< 0.001)	46.68 (< 0.001)	51.06 (< 0.001)	48.00 (< 0.001)	47.58 (< 0.001)	46.42 (< 0.001)	44.78 (< 0.001)
Race/ethnicity	181.24 (< 0.001)	172.30 (< 0.001)	180.74 (< 0.001)	179.81 (< 0.001)	179.57 (< 0.001)	179.26 (< 0.001)	178.73 (< 0.001)	75.42 (< 0.001)	506.12 (< 0.001)
SES	1.34 (0.261)	1.25 (0.287)	1.33 (0.263)	1.34 (0.263)	1.34 (0.261)	1.35 (0.260)	1.35 (0.258)	1.37 (0.255)	10.09 (< 0.001)
lfParticC	6.63 (0.010)	3.40 (0.065)	6.37 (0.012)	7.44 (0.006)	10.57 (0.001)	2.97 (0.085)	3.88 (0.049)	3.69 (0.055)	0.08 (0.772)
Female X race/ethnicity	1.16 (0.324)	1.18 (0.316)	1.19 (0.312)	1.19 (0.314)	1.16 (0.324)	1.19 (0.311)	1.19 (0.313)	1.13 (0.335)	1.69 (0.166)
Age group X race/ethnicity	13.48 (< 0.001)	14.09 (< 0.001)	13.57 (< 0.001)	13.54 (< 0.001)	13.73 (< 0.001)	14.00 (< 0.001)	13.91 (< 0.001)	11.96 (< 0.001)	12.38 (< 0.001)
Female X age group	2.49 (0.083)	3.15 (0.043)	2.59 (0.075)	2.61 (0.073)	2.49 (0.083)	2.52 (0.080)	2.56 (0.077)	0.84 (0.433)	0.36 (0.697)
Female X age group X race/ethnicity	2.35 (0.028)	2.14 (0.045)	2.30 (0.032)	2.32 (0.031)	2.37 (0.028)	2.20 (0.040)	2.22 (0.038)	2.12 (0.048)	1.04 (0.394)
*Temporal Effects*									
time (dummied)		19132.8 (< 0.001)							
time (linear/PWLT)	9.14 (0.003)		0.31 (0.577)	0.67 (0.412)	3.96 (0.047)	0.05 (0.826)	0.21 (0.643)	0.16 (0.688)	4.33 (0.037)
timeCC (PWLT)			0.69 (0.407)			7.61 (0.006)			
lagCC (PWLT)				0.57 (0.452)			14.82 (< 0.001)	15.94 (< 0.001)	14.06 (< 0.001)
time4CV (PWLT)					0.10 (0.751)	3.30 (0.069)	5.98 (0.015)	6.11 (0.013)	27.91 (< 0.001)
*Random effects (std err)*									
Census division: intercept	0.0895 (0.0319)	0.0898 (0.0325)	0.0896 (0.0319)	0.0895 (0.0318)	0.0894 (0.0317)	0.0892 (0.0317)	0.0890 (0.0316)	0.1569 (0.0573)	0.0664 (0.0321)
Race/ethnicity: intercept${}^{\text{*}}$								0.1015 (0.0393)	
Survey wave: intercept									0.0005 (0.0013)

*Notes* -2 Log L = model log likelihood; AIC = Akaike Information Criterion; BIC = Bayesian information criterion; std err = standard error; SES = socioeconomic status; lfParticC = census division labor force participation rate, mean centred within divisions; time = linear trend for time; PWLT = piecewise linear trend; timeCC = PWLT starting in 2008 when Camel Crush cigarettes were first introduced into the US market; lagCC = PWLT starting in 2010, allowing for a lag effect of the introduction of Camel Crush cigarettes, i.e., greater market penetration; time4CV = PWLT starting in 2016, corresponding to the first wave of the ITC 4CV Survey. ${}^{\text{*}}$ The random intercept for race/ethnicity was nested within census divisions

#### Validation

Modeled estimates were compared to external direct estimates from TUS-CPS for years in which both an ITC survey and TUS-CPS survey were conducted. Four sets of direct estimates of menthol use were generated for each census division within survey year using PROC CROSSTAB in SUDAAN: overall division-level prevalence, prevalence by sex, prevalence by age group, and prevalence by race/ethnicity. Confidence intervals were estimated using the replicate weights from TUS-CPS, setting Fay’s adjustment factor to 0.5 (i.e., ADJFAY = 4 in SUDAAN) [37]. The agreement between modeled and direct estimates was compared using the overall concordance correlation coefficient (oCCC) which includes components of both precision and accuracy [38–40]. Comparisons were conducted using the “epiR” library (Version 2.0.61) in R [41].

## Results

### Sample characteristics

Across 12 waves of the ITC US Survey conducted from 2002 to 2020, 12,020 adults who smoked at least monthly could be classified according to their use of menthol cigarettes. Of these, 18% were recruited in 2002, 15% were recruited in 2015, and 10% were recruited in each of 2016 and 2018 (Supplementary Table 2, Additional file 1). The remaining participants were recruited in all other survey years. About 60% of respondents participated in only a single wave of the ITC US Survey, with a greater percentage of respondents smoking menthol cigarettes participating in a single wave (64%) than respondents smoking non-menthol cigarettes (56%). A slightly greater percentage of participants were female (53%) than male (47%). The weighted distribution of respondents across the nine census divisions was similar to the overall distribution of the US population in 2020. Although most participants were non-Hispanic White, a much greater percentage of adults who smoked menthol cigarettes were non-Hispanic Black (24%) compared to those who smoked non-menthol brands (3%). Most participants (87%) smoked cigarettes daily at the time of recruitment into the ITC US Survey.

### Multilevel models

Table 1 presents fit statistics, tests of fixed effects, and random effects for the nine estimated multilevel logistic regression models. For all models, there was significant variation in the probability of smoking menthol cigarettes across census divisions, as indicated by the estimated variances and standard errors of the census division random intercept. Models that fit linear temporal trends (model 1) or a linear trend with a single bend in the trend line (models 3 and 5) showed similar levels of fit to the data, as suggested by AIC and BIC statistics. When time was fit as an indicator variable (model 2), there was some improvement in model fit. When two bends were allowed in the temporal trend (models 6 and 7), fit also improved slightly. However, when a nested random intercept was fit for race/ethnicity (model 8 AIC = 23,551.0), there was a noticeable improvement in model fit compared to models 6 and 7 (AIC = 23,608.9 and 23,607.2, respectively). An unweighted model (model 9) that fit a random intercept for census division and a second random intercept for time did not fit the data as well as the other models (AIC = 24,520.1).

Across all models, model 8 had the smallest value for both AIC and BIC statistics, suggesting this model was the best fitting model of the nine examined. Sex, age group, and race/ethnicity were significantly associated with the use of menthol cigarettes. There was also a significant three-way interaction between sex, age group, and race/ethnicity (*p* = 0.048). Although socioeconomic status was not significantly associated with the use of menthol cigarettes, it was retained in the model because it was included in the post-stratification frame used to estimate the prevalence of menthol use. The division-level labor force participation rate was also associated with using menthol cigarettes. Supplementary Table 3 (Additional file 1) presents estimated model parameters (log odds ratios) for model 8, the model selected to predict the prevalence of menthol cigarette use across census divisions for different demographic subgroups.

### Division-level trends in the prevalence of menthol cigarette use among adults who smoke

#### Overall trends

**Fig. 1:**
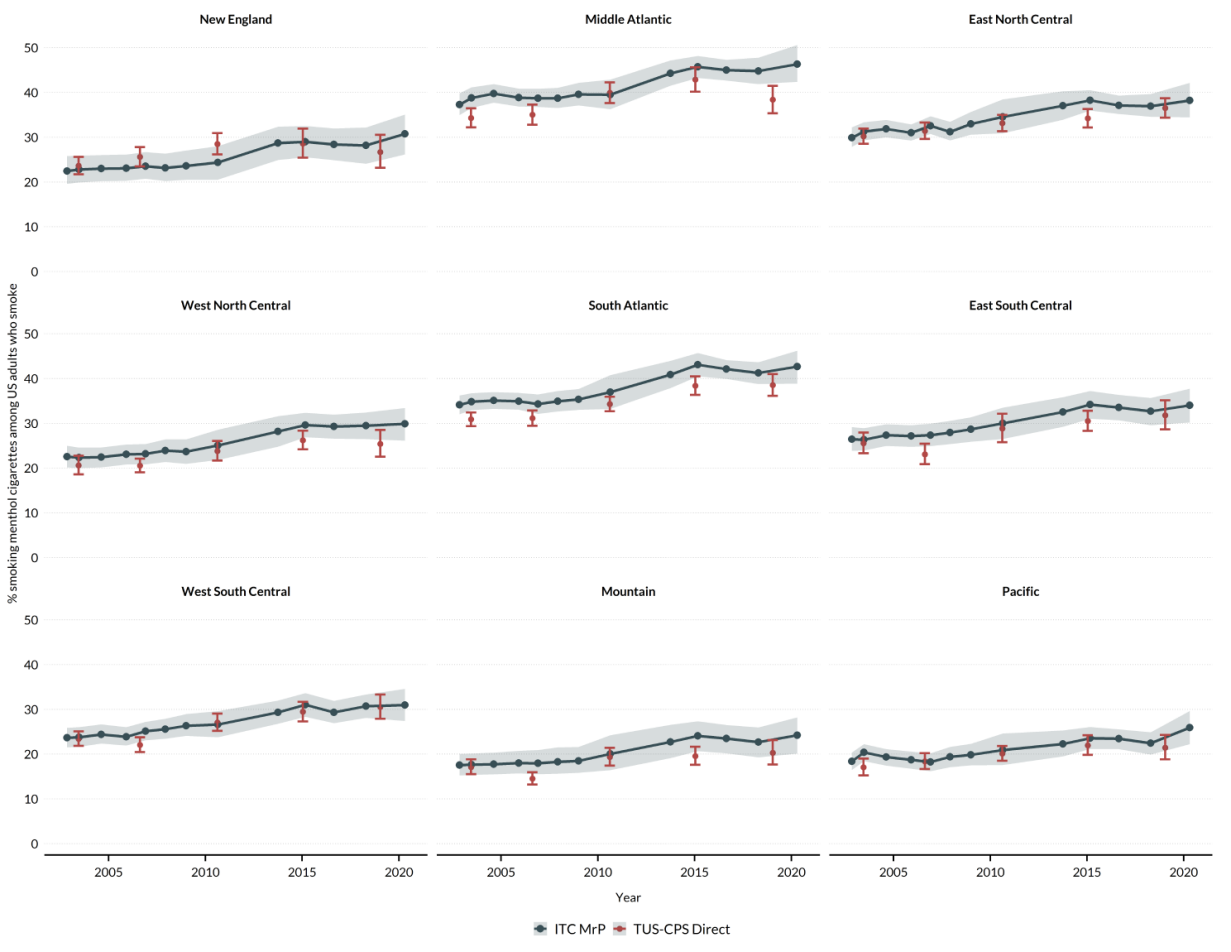
Overall prevalence of menthol cigarette use among US adults who currently smoke from 2002 to 2020 by census division. ITC MrP = modeled prevalence using the International Tobacco Control US data with multilevel regression and post-stratification. TUS-CPS Direct = direct survey estimates from the Tobacco Use Supplement to the Current Population Survey

There was a steady increase in the prevalence of menthol cigarette use among adults who smoked across all US census divisions from 2002 to 2020 (Fig. 1). There were also differences in estimated prevalence across census divisions: prevalence was highest in all years in the Middle Atlantic (2002 = 37.3%; 2020 = 46.3%) and South Atlantic (2002 = 34.1%; 2020 = 42.7%) divisions and lowest in the Mountain (2002 = 17.5%; 2020 = 24.2%) and Pacific (2002 = 18.4%; 2020 = 25.9%) divisions. Modeled estimates were generally similar to external, direct estimates from TUS-CPS, as suggested by the overlapping confidence intervals. However, there were some differences between modeled and direct estimates. For example, in the Middle Atlantic and South Atlantic divisions, modeled estimates were higher than direct estimates by an average of 3.4% points across all years. In the other divisions, modeled estimates were more similar to direct estimates, differing by an average of 0.9 (West South Central) to 2.6 (West North Central) percentage points across all years.

#### Trends by sex

Similar to the overall trend, the prevalence of menthol use among male and female adults who smoked increased in all census divisions from 2002 to 2020 (Supplementary Figs. 1 and 2, Additional file 1). These patterns were captured in both modeled estimates from ITC and direct estimates from TUS-CPS. Across divisions, the modeled prevalence of menthol use was, on average, 4.3 (South Atlantic) to 5.7 (East North Central) percentage points higher among females than males across all survey years. By 2020, more than 30% of females who smoked were smoking menthol brands across all areas of the US, except in the Mountain (27.0%) and Pacific (29.3%) divisions.

Modeled trends from ITC were more similar to direct estimates from TUS-CPS for females than males in most census divisions. Among females, modeled estimates were within 1.4% points compared to those from TUS-CPS in most census divisions. The exception was New England, where modeled prevalence was, on average, 3.6% points lower than estimates from TUS-CPS. Among males, modeled estimates were 5.1 and 6.4% points higher than those from TUS-CPS in the South Atlantic and Middle Atlantic divisions. Modeled estimates among males were more similar to those from TUS-CPS in the New England, East South Central, and West South Central divisions.

#### Trends by age group

Menthol use among adults who smoked was most prevalent among those aged 18–29 and least common among those aged 50 and older (Supplementary Fig. 3 through 5, Additional file 1). In most divisions, the modeled prevalence of menthol use exceeded 30% in all survey years for adults aged 18–29. Even in the Mountain and Pacific divisions, prevalence of use exceeded 30% in this age group by 2020. Although modeled trends generally mirrored those from TUS-CPS for all age groups, modeled estimates were, on average, 1.6 (South Atlantic) to 7.5 (West North Central) percentage points higher than those from TUS-CPS for adults aged 18–29. Differences between modeled estimates and direct estimates were smaller among adults aged 50 and older. In that age group, modeled estimates were, on average, lower than those from TUS-CPS in most census divisions except in the Middle and South Atlantic. Direct estimates from TUS-CPS suggest that prevalence of use had declined by 2019 among those aged 18–29 from the New England, Middle Atlantic, West North Central, and Pacific divisions.

#### Trends by race/ethnicity

Both modeled and direct estimates of prevalence varied most by race/ethnicity (Supplementary Fig. 6 through 9, Additional file 1). The modeled prevalence of menthol use was highest among non-Hispanic Black adults who smoked in all years across all census divisions (Supplementary Fig. 7, Additional file 1). There was also less fluctuation in modeled estimates across survey years compared with direct estimates for non-Hispanic Black adults. Moreover, modeled estimates tended to overestimate direct estimates of prevalence in this demographic. There was also greater uncertainty about estimated prevalence, as indicated by the wider confidence intervals for both modeled and direct estimates. This was true for all race/ethnic groups in most census divisions except among non-Hispanic White adults.

Hispanic adults who smoked had the greatest variability in prevalence of menthol use across census divisions. In the Middle Atlantic, modeled prevalence was 71.3% in 2020 (2019 TUS-CPS = 50.7%), approaching that of non-Hispanic Black adults (2020 modeled = 85.6%; 2019 TUS-CPS = 75.7%). In other divisions, however, both modeled and direct estimates of prevalence were more similar to those among non-Hispanic White adults (e.g., Pacific 2020: Hispanic = 26.5%, non-Hispanic White = 19.3%). Among adults from all “other” race/ethnic groups, the modeled prevalence of menthol use in the West South Central, Mountain, and Pacific divisions was similar to prevalence among non-Hispanic White adults who smoke in all years.

#### Agreement between modeled and direct estimates

The overall concordance correlation coefficient was used to assess agreement between modeled and direct estimates for each demographic subgroup (i.e., overall, by sex, by age group, and by race/ethnicity). Agreement is presented in Supplementary Fig. 10 (Additional file 1) as scatterplots that plot modeled estimates from ITC against direct estimates from TUS-CPS. The red line represents the line of best fit, estimated by regressing modeled estimates on direct estimates. It measures the precision of the estimates, where less spread about the line reflects greater precision. The black line represents the concordance line, or line of perfect agreement. The closer the line of best fit to the concordance line, the greater the level of agreement between the two sets of estimates (i.e., a concordance correlation of 1 represents perfect agreement).

Division-specific modeled estimates of the prevalence of menthol use among adults who smoke showed relatively high agreement with direct estimates from TUS-CPS (overall concordance correlation = 0.932, agreement = 0.966, precision = 0.965). Agreement was also high (> 0.91) for estimates among females and among non-Hispanic white adults. Agreement was lower for males and for age-specific estimates (oCCC > 0.81). The shift in the line of best fit relative to the concordance line for all these groups suggests that modeled estimates generally over-estimated prevalence compared to TUS-CPS. Modeled estimates for non-Hispanic black, Hispanic, and adults from all other race/ethnic groups showed low levels of agreement compared with those from TUS-CPS (oCCC = 0.55, 0.70, and 0.52, respectively).

Supplementary Table 4 (Additional file 1) compares agreement between modeled and direct estimates across the nine multilevel models. Consistent with AIC and BIC fit statistics, group-specific estimates from model 8 showed the greatest level of agreement with estimates from TUS-CPS. In other words, model 8 had the highest values of the oCCC for 6 of 10 group-specific estimates (overall, males, adults aged 18–29, adults aged 30–49, non-Hispanic Black adults, and Hispanic adults).

#### Disparities in modeled prevalence by race/ethnicity

**Fig. 2:**
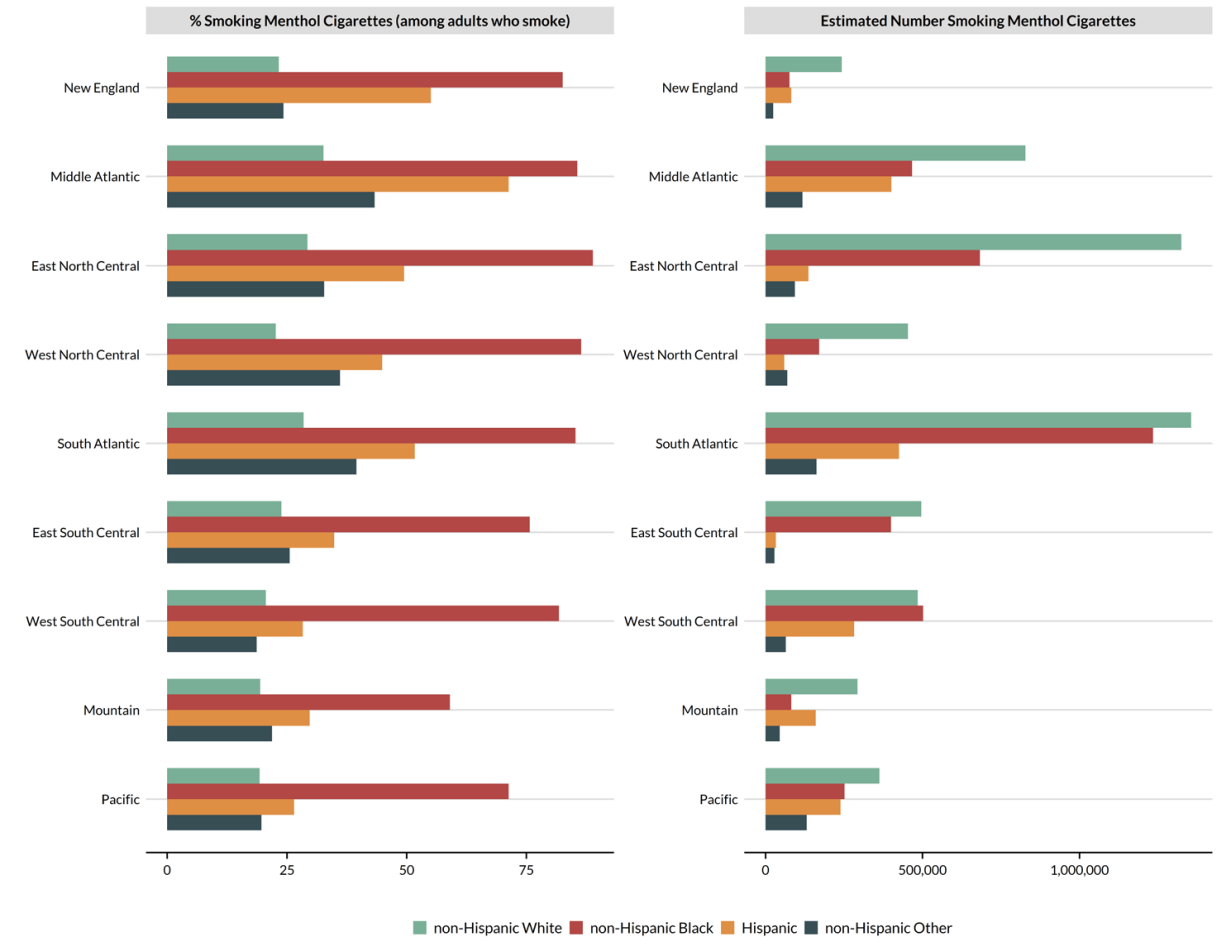
Disparities in the prevalence of menthol cigarette use among adults who smoke by race/ethnicity in the United States in 2020 (ITC MrP modeled estimates)

Figure 2 presents differences in the modeled prevalence of menthol use by race/ethnicity in 2020 across census divisions. The left panel of Fig. 2 presents the percentage of adults using a menthol brand among those who smoke whereas the right panel presents the estimated number of adults smoking menthol cigarettes. Across all census divisions, menthol use was most prevalent among non-Hispanic Black and Hispanic adults who smoked and least common among non-Hispanic White adults who smoked. However, the demographic composition of different areas of the US and the prevalence of smoking within those groups influences the total number of adults smoking menthol cigarettes. This is demonstrated in the right panel of Fig. 2. Based on modeled estimates from ITC, in the Middle Atlantic states, more than 800,000 non-Hispanic White adults smoked menthol cigarettes in 2020. However, a greater number of adults from all other race/ethnic groups combined smoked menthol cigarettes in 2020. In the South Atlantic, nearly as many non-Hispanic Black adults smoked menthol cigarettes as non-Hispanic White adults. This was also true in the East South Central and West South Central census divisions.

## Discussion

This study found that the prevalence of menthol cigarette use among adults who smoke increased from 2002 to 2020 in the United States. Unlike previous studies [5, 6], this study disaggregated trends in the prevalence of menthol cigarette use by US census division and by demographic subgroups within divisions. Geographic trends in prevalence varied in ways consistent with the past regional targeted marketing practices of cigarette companies [1, 2]. Across all nine divisions, the prevalence of menthol cigarette use among adults who smoked increased over the 18-year study period. These trends are consistent with changes in regional trends noted by Seaman et al. [5]. In addition, prevalence of use varied across divisions, such that by 2020, 24% (Mountain division) to 46% (Middle Atlantic division) of adults who smoked were smoking menthol cigarettes.

Increases in the use of menthol cigarettes were evident for most demographic subgroups in all census divisions. Although increases in prevalence among non-Hispanic Black adults were slightly smaller than among all other racial/ethnic groups, because prevalence increased in all groups within all divisions, disparities in the prevalence of use between groups changed little over time. As such, substantial disparities in menthol cigarette use remain, especially between non-Hispanic White adults and non-Hispanic Black adults.

Important differences in the prevalence of menthol use were also observed by sex and by age group. Menthol use was higher across all divisions among adult females who smoked over the course of the study than among adult males who smoked. Differences between males and females, however, tended to be smaller than differences between the youngest and oldest age groups. For example, in the Middle Atlantic division, 57.3% of adults ages 18–29 who smoked were smoking menthol cigarettes in 2020, compared to 41% of adults ages 50 or older. Similar differences in prevalence were observed In the New England, East North Central, South Atlantic, and East South Central divisions.

Disparities in the prevalence of menthol cigarette use among adults who smoke translate into important differences in the estimated number of adults smoking menthol cigarettes, as shown in Fig. 2. It is useful to note that even though the prevalence of menthol use among non-Hispanic Black adults who smoke was much higher than among all other race/ethnic groups, the demographic composition of the population influences the estimated number of adults smoking menthol cigarettes within a given area. For example, in the South Atlantic division in 2020, 28% of non-Hispanic White adults who smoked used menthol cigarettes compared to 85% of non-Hispanic Black adults who smoked (Fig. 2). This translates to nearly equal numbers of people from both groups smoking menthol cigarettes. Considering all minority groups, the total number of people smoking menthol cigarettes exceeds the total number of non-Hispanic White adults smoking menthol cigarettes in many census divisions. Thus, eliminating menthol from cigarettes has significant potential to reduce differences in the burden of disease from smoking for minority populations, thereby advancing health equity [7].

### Strengths and limitations

Using multilevel regression and post-stratification, this study estimated long-term trends in the prevalence of menthol cigarette use among adults who smoke at sub-national levels for specific demographic groups. These refined estimates provide a nuanced picture of long-term trends in menthol cigarette use across different areas of the US. One important strength of this study is the comparison of modeled trends using data from the ITC US Project against direct, survey-based estimates from the nationally representative TUS-CPS. Modeled estimates were generally consistent with direct estimates for most demographic groups. However, it is important to note that modeled estimates showed lower agreement with direct estimates for non-Hispanic Black adults, Hispanic adults, and adults from all other race/ethnic groups combined. Compared to direct estimates in these groups, modeled estimates from ITC overestimated prevalence. However, this does not necessarily mean that modeled estimates are inferior to those from TUS-CPS: division-specific direct estimates among minority groups often had confidence intervals as wide as modeled estimates from ITC. Even though the overall sample size of TUS-CPS for any given survey wave was large—exceeding 16,000 respondents in any given wave—after slicing the data by census division and race/ethnicity, available sample sizes for estimating prevalence of menthol use among adults who smoke were much smaller, leading to greater uncertainty and wider confidence intervals around direct estimates.

Another limitation of the modeled estimates is that they may have over-estimated the prevalence of menthol cigarette use among adults who smoke compared to those from TUS-CPS. For example, among non-Hispanic Black adults, the modeled prevalence of menthol use exceeded 80% in many census divisions whereas estimates from TUS-CPS were typically lower. However Goodwin et al. [6], found that the prevalence of menthol use among this demographic at the national level was 84.9% in 2019 using data from NSDUH. Thus, division-level modeled estimates from ITC may be a reasonable representation of the the prevalence of menthol use within different demographic subgroups.

Differences in the measurement of menthol cigarettes use between the ITC and TUS-CPS surveys may have also contributed to differences between modeled and direct estimates. Modeled estimates from ITC were based on last purchased brand of cigarettes and usual brand of cigarettes, if usual brand differed from last purchased brand. If either brand was identified as a menthol brand, ITC respondents were classified as smoking menthol cigarettes. In TUS-CPS, use of menthol cigarettes was defined using a single question only. A sensitivity analysis that classified menthol use for ITC respondents according to last purchased brand only showed small improvements in agreement between modeled and direct estimates. This was true for the overall prevalence of menthol use and all subgroup estimates except those among (a) females and (b) all other race/ethnic groups combined.

It should also be noted that the fitted multilevel models used the cross-sectional survey weights from each wave of the ITC US Survey. The modeling also did not account for repeated measures arising from respondents participating in more than one survey wave. However, because the purpose of the modeling was to estimate proportions at the census division level rather than model relationships among those proportions, the standard errors for the division-level estimates that ignore the repeated measures structure and assume independent observations from wave to wave are likely conservative.

Another consideration relates to the modeling approach itself: data from twelve ITC Survey waves were used to estimate longitudinal trends in the use of menthol cigarettes. It is possible that the estimated multilevel model borrowed too much strength across multiple census divisions and survey waves, producing division-specific trends that were too similar to the overall average trend. However, the modeling approach used here highlights the ability to examine long-term sub-national trends in the prevalence of menthol cigarettes use among US adults who smoked from 2002 to 2020. Estimated trends suggested that disparities in the prevalence of menthol use between demographic groups changed little during this time period.

It is also important to point out that methodological changes were made to the BRFSS in 2011 that may influence direct estimates of health risk behavior prevalence, impeding comparison of estimates across years [18]. Specifically, the addition of a cellular telephone sampling frame may have increased coverage of people (a) from younger age groups, (b) having lower incomes, and (c) having lower educational levels [18]. Increased coverage of these groups affects estimates of health risk behaviors because these behaviors are more common in these groups. However, in this study, the BRFSS data were used to estimate the total number of people who smoke in 72 cross-classified demographic groups within census divisions. The multilevel modeling strategy controlled for factors on which the cellular telephone and other parts of the BRFSS frame differ across survey years. Furthermore, modeled estimates were similar to direct estimates from TUS-CPS across survey years, suggesting that the methodological changes to BRFSS may not have biased these model-based estimates to the same extent as direct estimates from BRFSS.

A final limitation that merits attention is that data for the 2020 ITC Survey wave were collected during the initial outbreak of COVID-19 in the US. While it is possible that smoking behaviors may have been affected by the pandemic, a previous analysis of the ITC US data compared carton purchases and cigarette consumption in 2020 during three calendar periods (before March 19, March 19–April 23, after April 23) to those same periods in 2018 [42]. This study found that although the percentage of people who purchased cigarettes by the carton was significantly higher in 2020 than in 2018, average cigarette consumption did not differ during any of the three calendar periods in either year. While people who smoke may have stockpiled cigarettes during the initial outbreak of COVID-19, they did not seem to change their smoking behaviors [42–44]. Thus, the impact of the pandemic on the prevalence of menthol cigarette use among adults who smoke may have been minimal.

## Conclusion

The proposed US FDA menthol ban will exert different effects across geographic and demographic subgroups, depending on the demographic composition of the population of adults who smoke in different areas of the US. The FDA’s proposed ban on menthol cigarettes will differentially impact geographic regions of the US helping to reverse the targeted marketing of menthol cigarettes that has disproportionately impacted Black and Hispanic populations. Moreover, menthol bans promote smoking cessation [45]. Subgroups that smoke menthol at higher rates are expected to experience greater reductions in smoking prevalence following the ban [46], thereby reducing smoking-attributable health disparities between groups. Reductions in smoking prevalence, however, rely on access to effective smoking cessation services; the expected demand for those services may vary by geography and demographic group. States, therefore, should plan how to accommodate anticipated needs for cessation services prior to implementation of the FDA menthol ban.

### Electronic supplementary material

Below is the link to the electronic supplementary material.

Supplementary Material 1

## Data Availability

In each country participating in the International Tobacco Control Policy Evaluation (ITC) Project, the data are jointly owned by the lead researcher(s) in that country and the ITC Project at the University of Waterloo. Data from the ITC Project are available to approved researchers two years after the date of issuance of cleaned data sets by the ITC Data Management Centre. Researchers interested in using ITC data are required to apply for approval by submitting an International Tobacco Control Data Repository (ITCDR) request application and subsequently to sign an ITCDR Data Usage Agreement. The criteriafor data usage approval and the contents of the Data Usage Agreement are described on the ITC Project website. All other datasets used for this research were publicly available (the US Behavioral Risk Factor Surveillance System, the American Community Survey, and the Tobacco Use Supplement to the Current Population Survey).

## Abbreviations

ACS: American Community Survey
AIC: Akaike information criterion
BIC: Bayesian information criterion
BRFSS: Behavioral Risk Factor Surveillance System
CATI: Computer-assisted telephone interviewing
CAPI: Computer-assisted personal interviewing
CDC: Centers for Disease Control and Prevention
COVID-19: coronavirus disease 2019
CPS: Current Population Survey
FDA: Food and Drug Administration
ITC: International Tobacco Control
ITC 4C: International Tobacco Control Four Country Survey
ITC 4CV: International Tobacco Control Four Country Smoking and Vaping Survey
MrP: multilevel regression and post-stratification
NSDUH: National Survey on Drug Use and Health
oCCC: overall concordance correlation coefficient
PWLT: piecewise linear trends
SES: socioeconomic status
TUS-CPS: Tobacco Use Supplement to the Current Population Survey
US: United States
