# Supplementary material for: Prevalence of menthol cigarette use among adults who smoke from the United States by census division and demographic subgroup, 2002–2020: findings from the International Tobacco Control (ITC) project
Source: Popul Health Metr. 2024 Apr 9;22:6. doi: 10.1186/s12963-024-00326-0 (PMC11005135; doi:10.1186/s12963-024-00326-0)
Supplement: Supplementary file 1 — Supplementary Material 1 [file 12963_2024_326_MOESM1_ESM.pdf]

Supplementary Figures

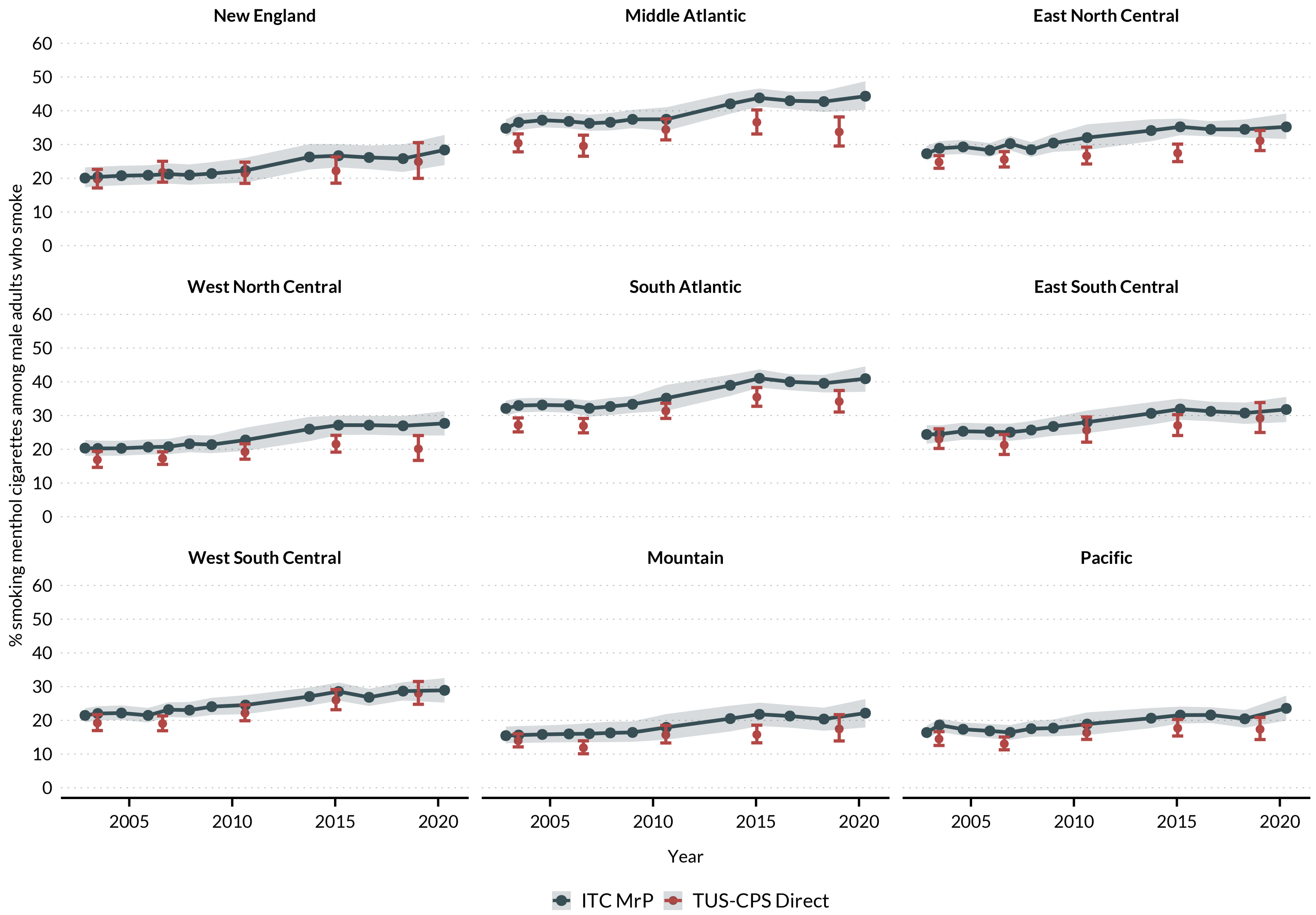

Figure 1. Prevalence of menthol cigarette use among male adults in the US who currently smoke from 2002 to 2020 by census division. ITC MrP = modeled prevalence using the International Tobacco Control US data with multilevel regression and post-stratification. TUS-CPS Direct = direct survey estimates from the Tobacco Use Supplement to the Current Population Survey.

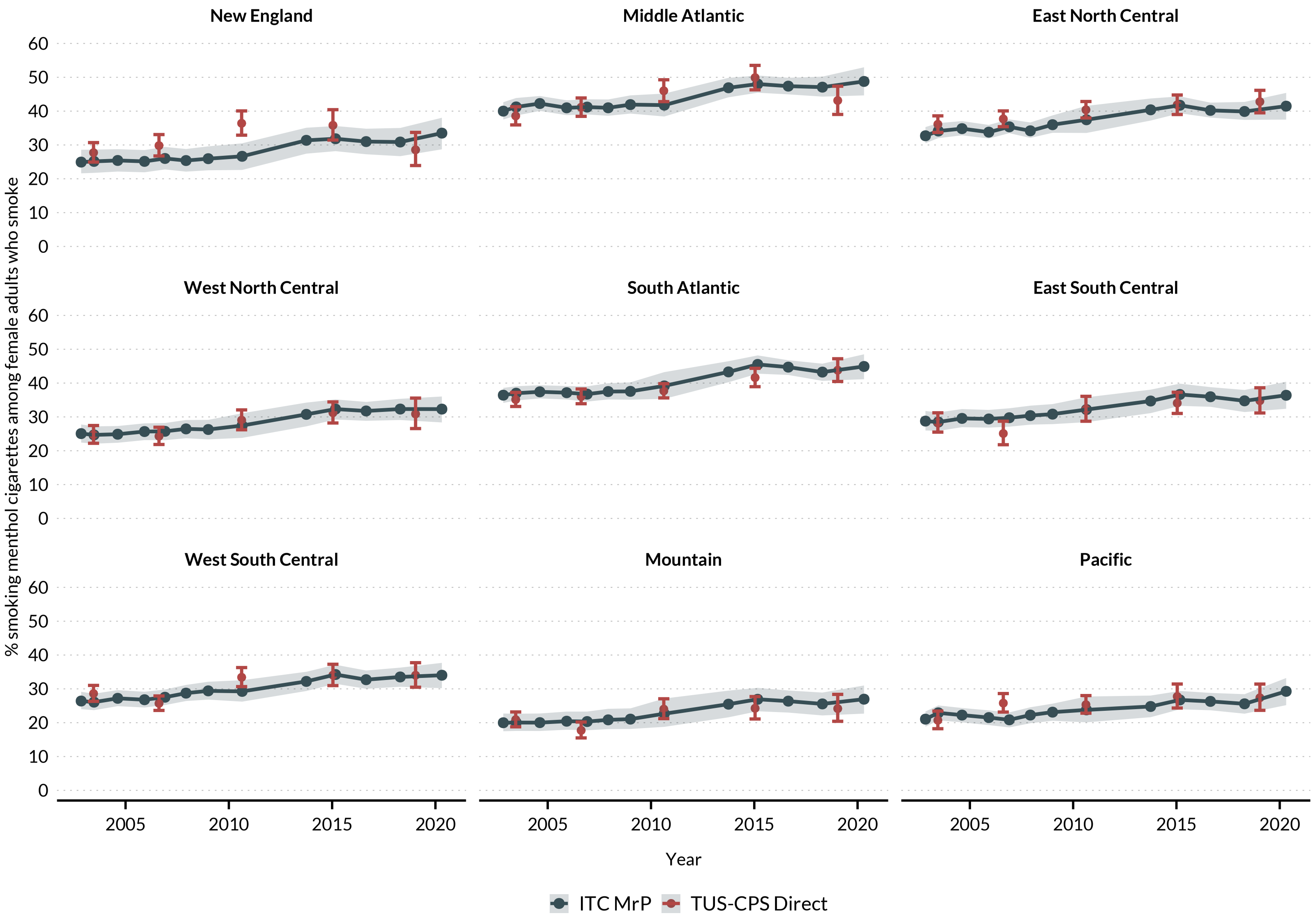

Figure 2. Prevalence of menthol cigarette use among female adults in the US who currently smoke from 2002 to 2020 by census division. ITC MrP = modeled prevalence using the International Tobacco Control US data with multilevel regression and post-stratification. TUS-CPS Direct = direct survey estimates from the Tobacco Use Supplement to the Current Population Survey.

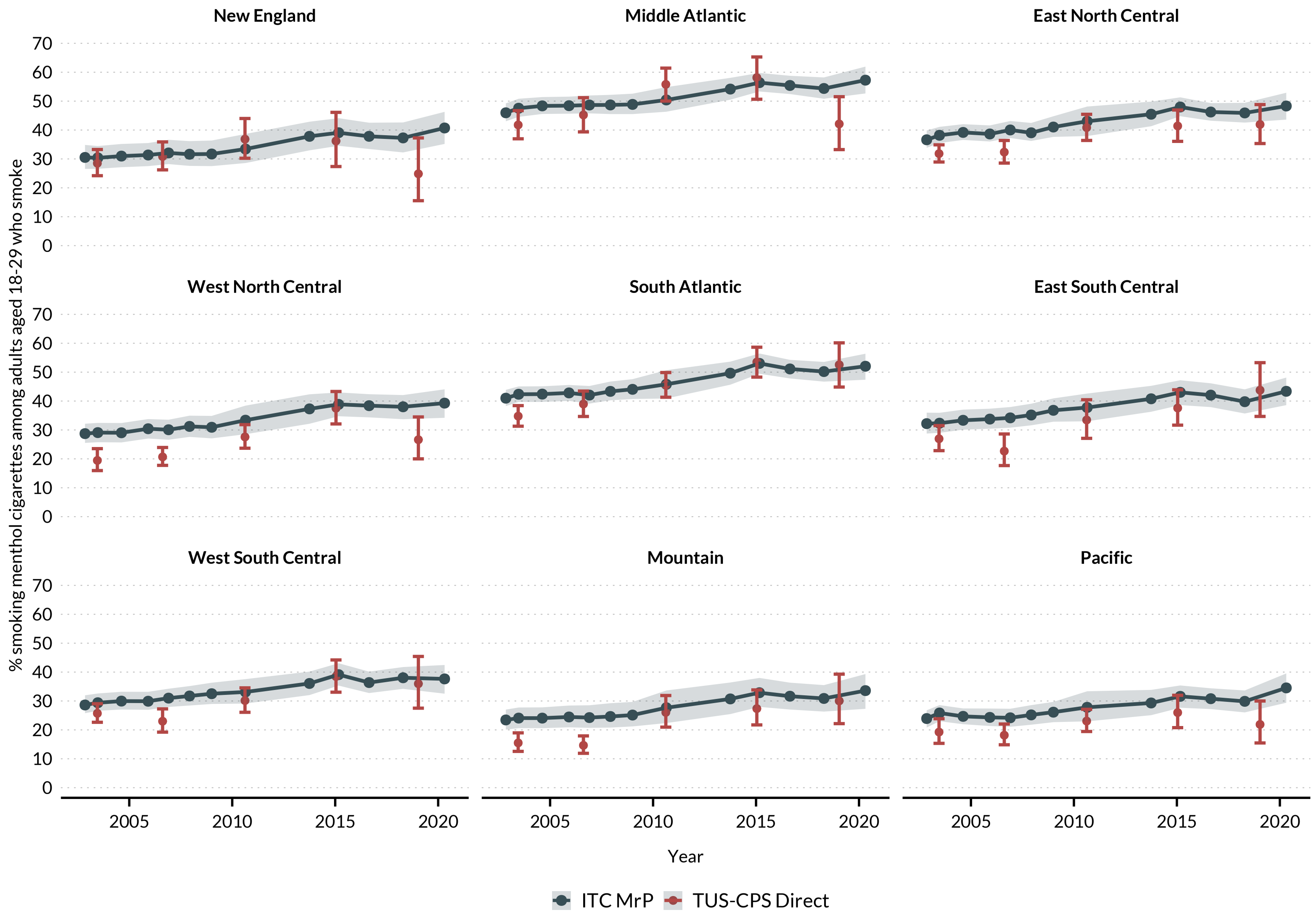

Figure 3. Prevalence of menthol cigarette use among adults in the US aged 18 to 29 who currently smoke from 2002 to 2020 by census division. ITC MrP = modeled prevalence using the International Tobacco Control US data with multilevel regression and post-stratification. TUS-CPS Direct = direct survey estimates from the Tobacco Use Supplement to the Current Population Survey.

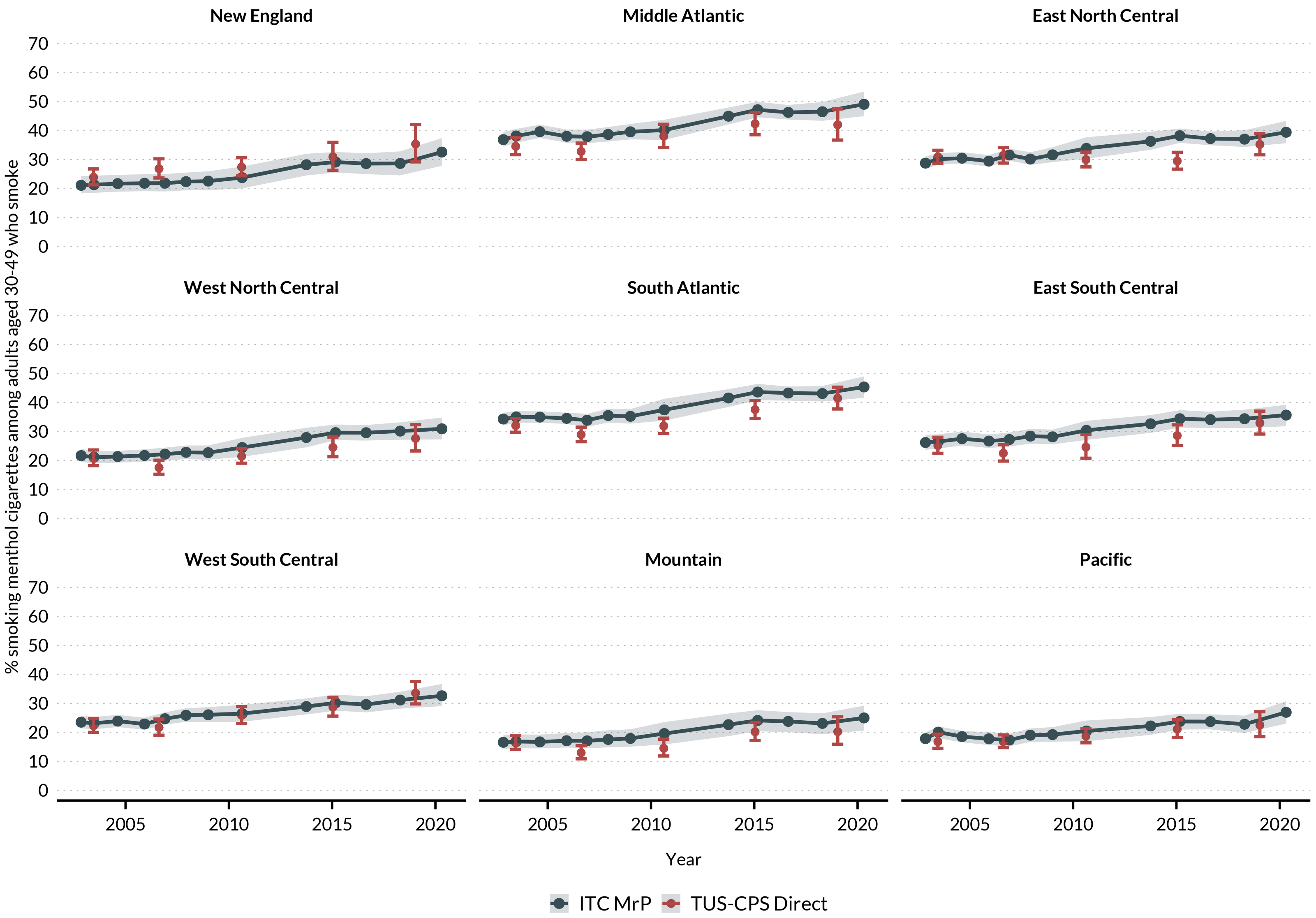

Figure 4. Prevalence of menthol cigarette use among adults in the US aged 30 to 49 who currently smoke from 2002 to 2020 by census division. ITC MrP = modeled prevalence using the International Tobacco Control US data with multilevel regression and post-stratification. TUS-CPS Direct = direct survey estimates from the Tobacco Use Supplement to the Current Population Survey.

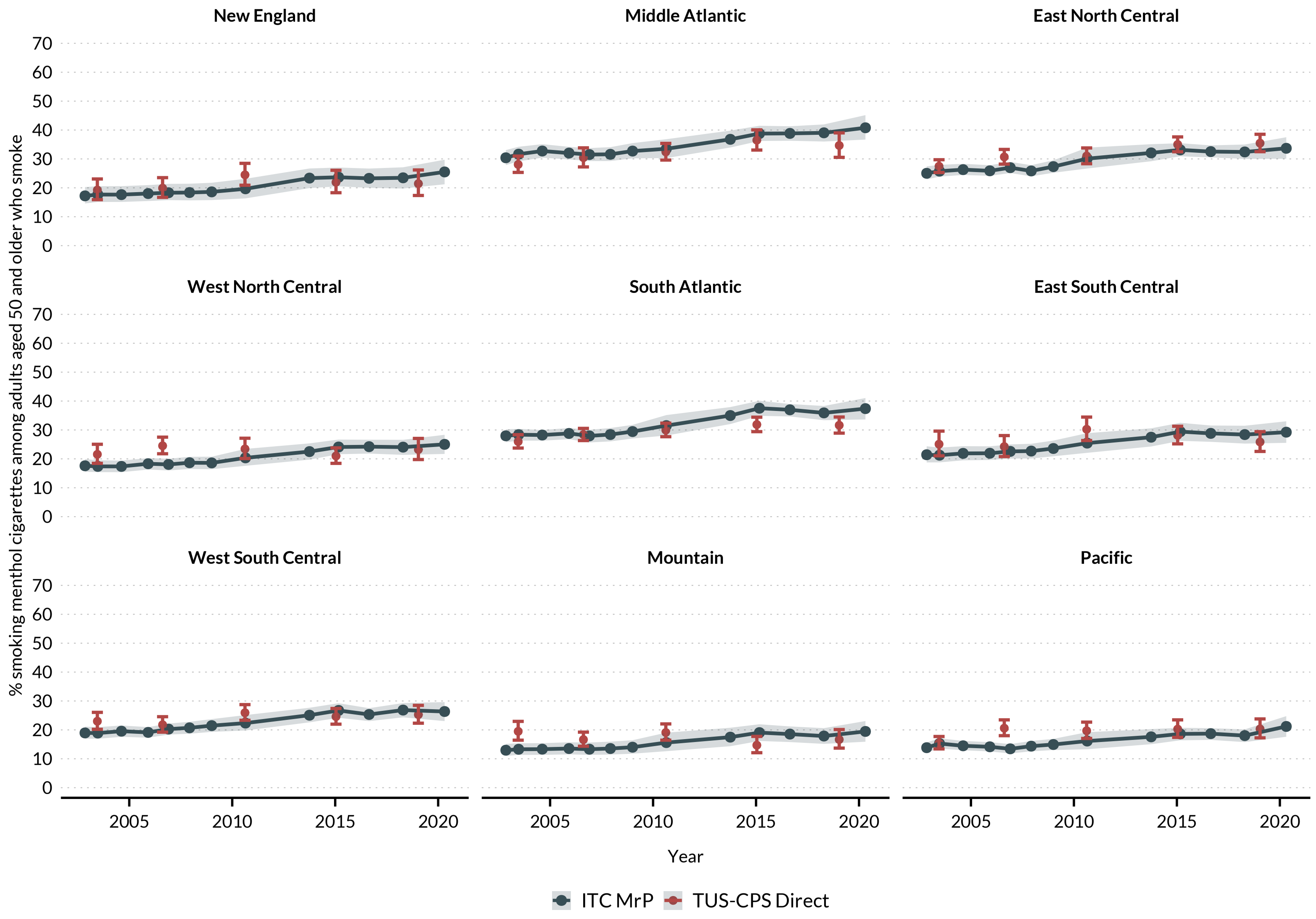

Figure 5. Prevalence of menthol cigarette use among adults in the US aged 50 and older who currently smoke from 2002 to 2020 by census division. ITC MrP = modeled prevalence using the International Tobacco Control US data with multilevel regression and post-stratification. TUS-CPS Direct = direct survey estimates from the Tobacco Use Supplement to the Current Population Survey.

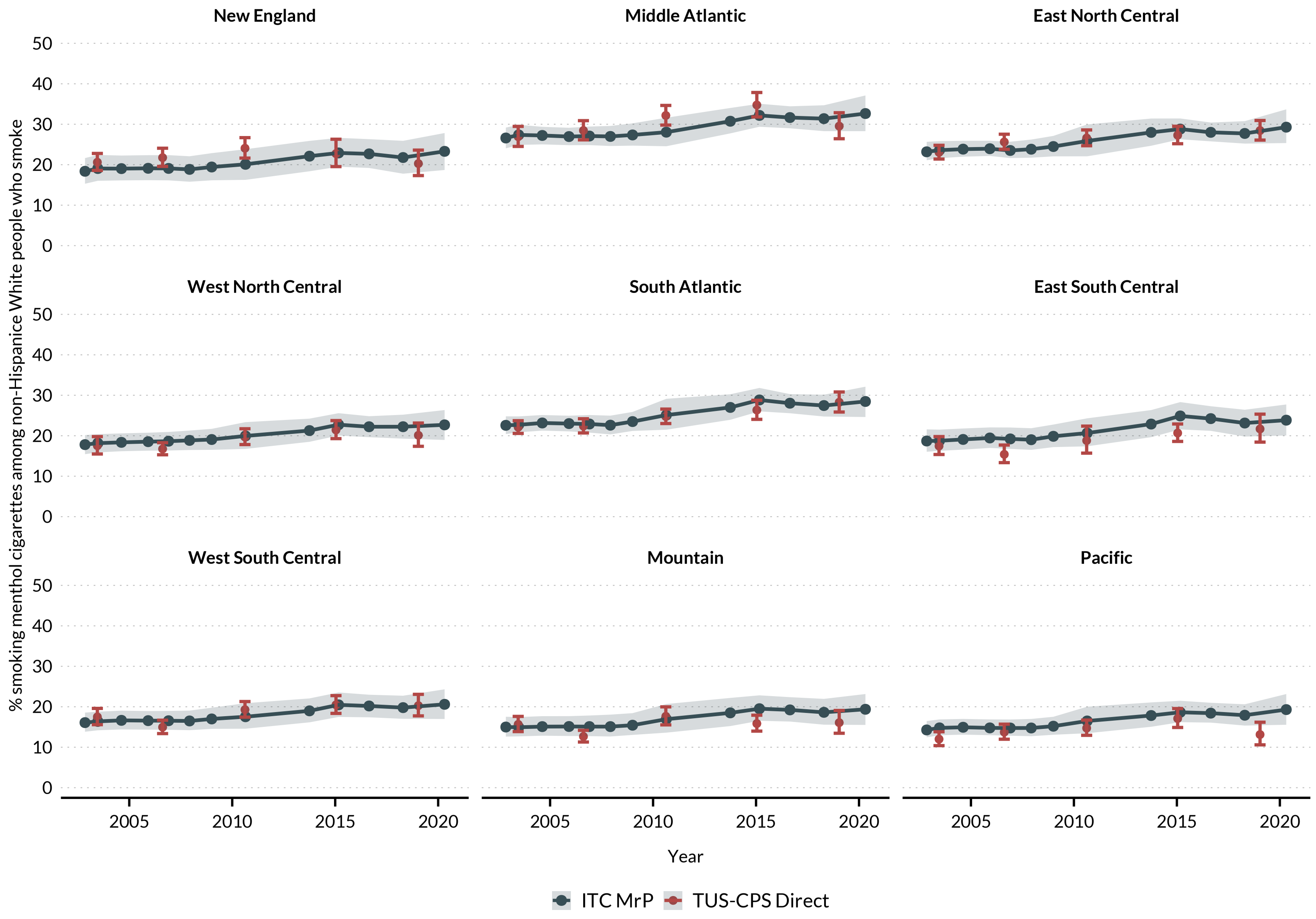

Figure 6. Prevalence of menthol cigarette use among non-Hispanic White adults in the US who currently smoke from 2002 to 2020 by census division. ITC MrP = modeled prevalence using the International Tobacco Control US data with multilevel regression and post-stratification. TUS-CPS Direct = direct survey estimates from the Tobacco Use Supplement to the Current Population Survey.

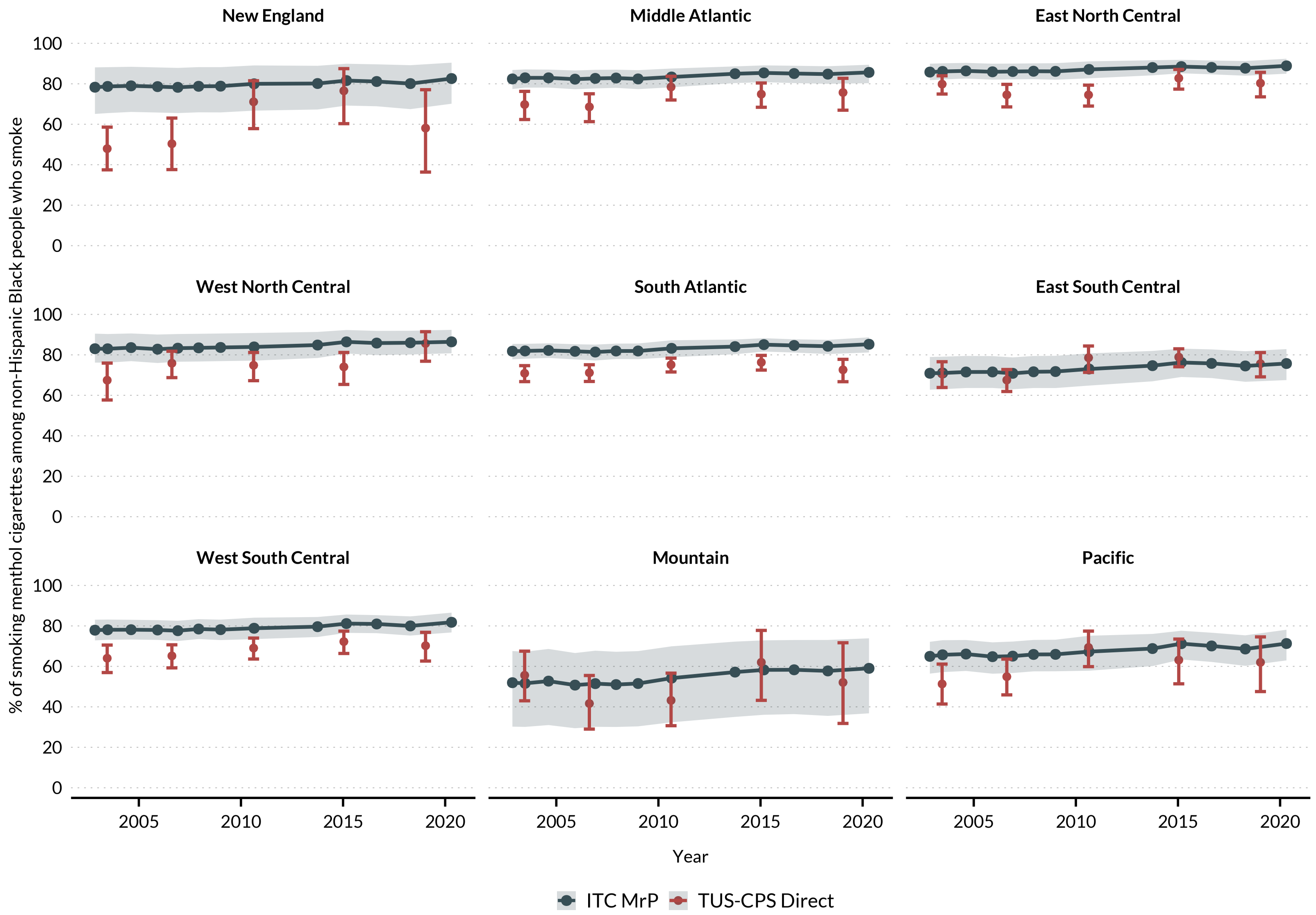

Figure 7. Prevalence of menthol cigarette use among non-Hispanic Black adults in the US who currently smoke from 2002 to 2020 by census division. ITC MrP = modeled prevalence using the International Tobacco Control US data with multilevel regression and post-stratification. TUS-CPS Direct = direct survey estimates from the Tobacco Use Supplement to the Current Population Survey.

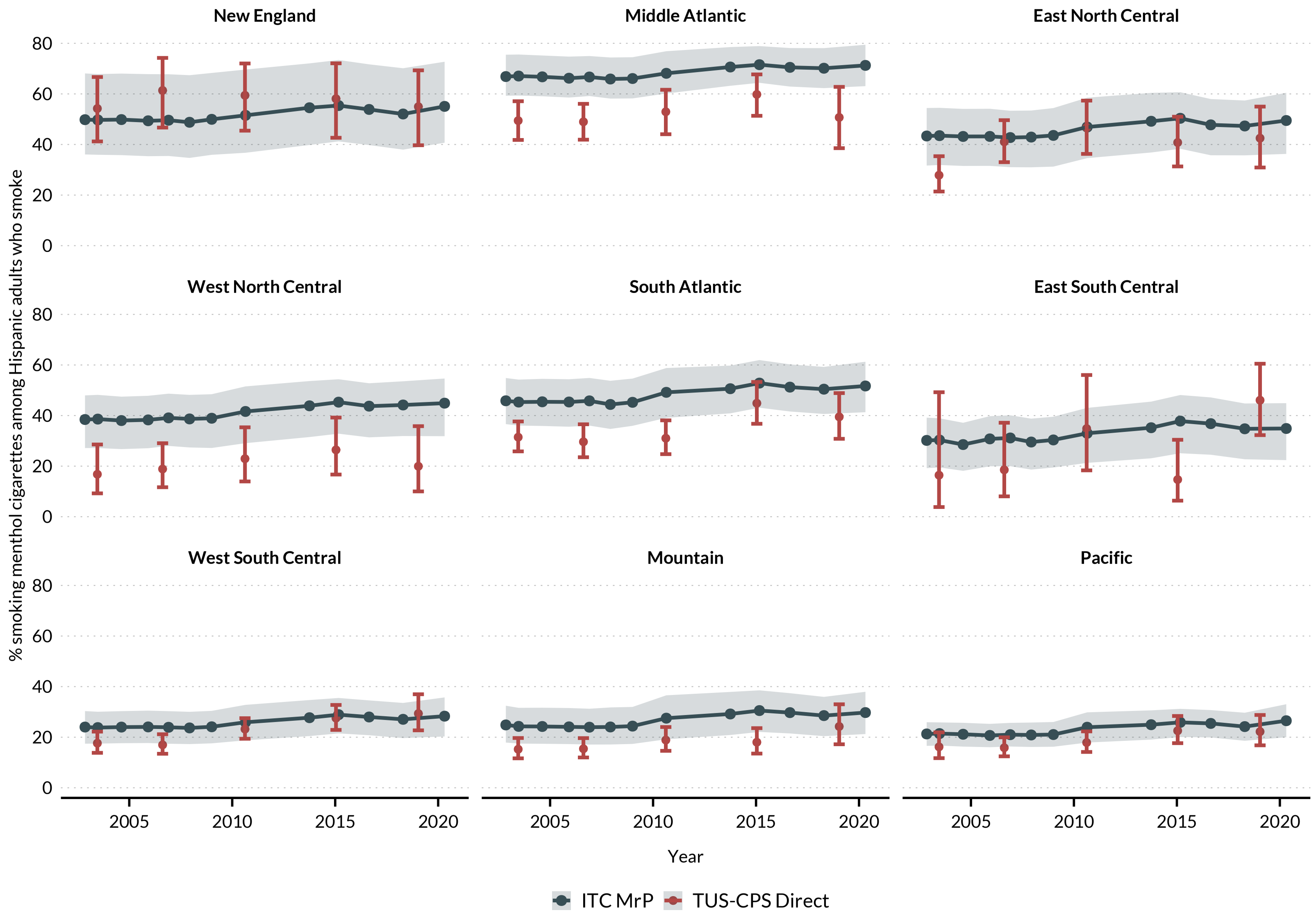

Figure 8. Prevalence of menthol cigarette use among Hispanic adults in the US who currently smoke from 2002 to 2020 by census division. ITC MrP = modeled prevalence using the International Tobacco Control US data with multilevel regression and post-stratification. TUS-CPS Direct = direct survey estimates from the Tobacco Use Supplement to the Current Population Survey.

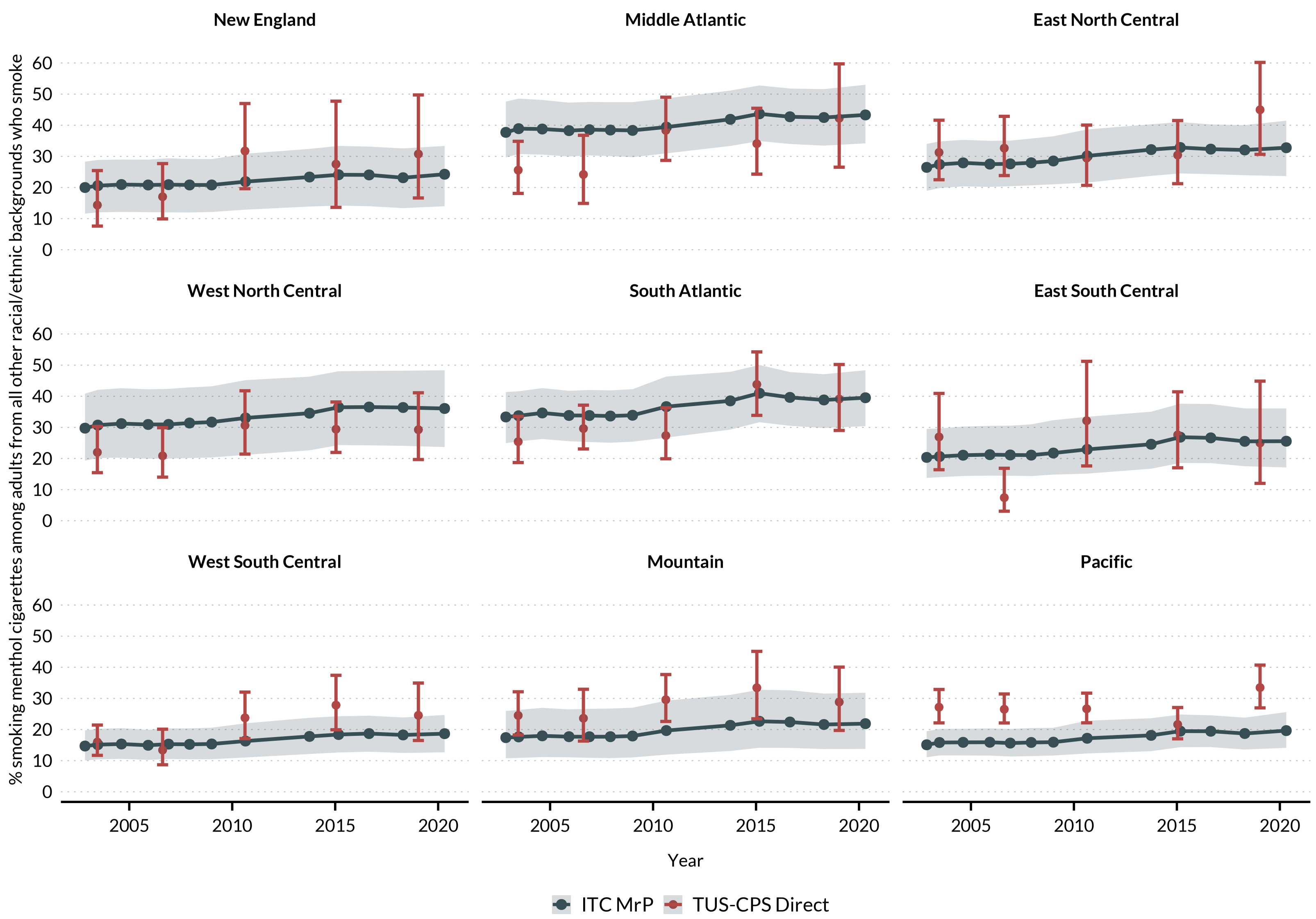

Figure 9. Prevalence of menthol cigarette use among adults from other racial/ethnic groups in the US who currently smoke from 2002 to 2020 by census division. ITC MrP = modeled prevalence using the International Tobacco Control US data with multilevel regression and post-stratification. TUS-CPS Direct = direct survey estimates from the Tobacco Use Supplement to the Current Population Survey.

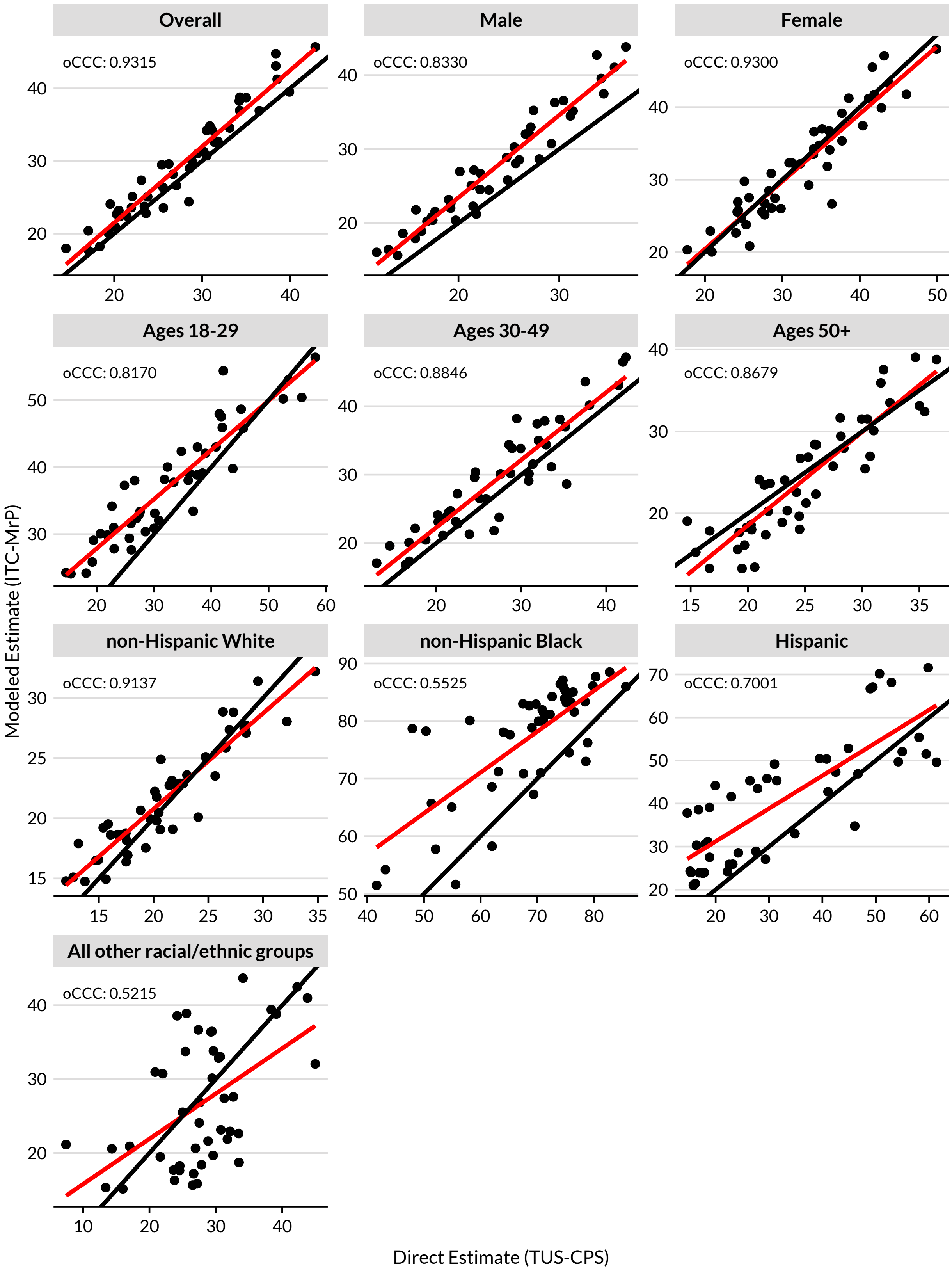

Figure 10. Agreement between modeled estimates of menthol cigarette use among current smokers (ITC MrP) and direct estimates from TUS-CPS using the best-fitting multilevel model (Model 8 in Supplementary Table 4).

Supplementary Tables

Supplementary Table 1. Data sources used for multilevel regression and post-stratification modeling of menthol cigarette use among US adults who smoke, 2002 to 2020.

Data Source	Design	Survey Wave	Survey Dates	Survey Mode	Sample Size
ITC 4C	stratified	Wave 1	Oct to Dec 2002	CATI	2102
	post-stratification	Wave 2	May to Sep 2003	CATI	1896
	weights	Wave 3	Jun to Dec 2004	CATI	1924
		Wave 4	Oct 2005 to Jan 2006	CATI	1792
		Wave 5	Oct 2006 to Feb 2007	CATI	1790
		Wave 6	Sep 2007 to Feb 2008	CATI	1744
		Wave 7	Oct 2008 to Feb 2009	CATI & Web	1518
		Wave 8	Jul 2010 to Jun 2011	CATI & Web	1262
		Wave 9 (a)	Aug 2013 to Oct 2014	CATI & Web	769
		Wave 9 (b)	Feb to May 2015	Web	1842
ITC 4CV	stratified	Wave 1	Jul to Sep 2016	Web	2190
	raking weights	Wave 2	Feb to Jul 2018	Web	2061
		Wave 3	Feb to Jun 2020	Web	1813
BRFSS	stratified	2002	Jan to Dec	CATI (landline)	240,735
	post-stratification	2003			257,659
	wights until 2010	2004			296,971
		2005			349,901
		2006			347,790
		2007			423,783
		2009			406,749
		2010			444,927
	raking weights	2013	Jan to Dec	CATI (landline	483,865
	since 2011	2015		& mobile)	434,382
		2016			477,665*
		2018			430,949†
		2020			394,831
ACS	multistage cluster	2002	12 monthly independent	Mail, internet	809,627
	replicate weights	2003	samples (data collection	CATI, CAPI	902,194
	raking weights	2004	spans a three-month		901,746
		2005	period)		2,173,772
		2006			2,187,662
		2007			2,214,156
		2009			2,226,602
		2010			2,291,701
		2013			2,317,301
		2015			2,348,374
		2016			2,360,775
		2018			2,414,823
		2020			1,957,499
TUS-CPS	multistage cluster	Wave 6	Feb, Jun, Nov 2003	CATI & CAPI	183,810 (34,644)‡
	replicate weights	Wave 7	May, Aug 2006; Jan 2007		172,023 (31,501)‡
		Wave 8	May, Aug, 2010; Jan 2011		171,365 (27,611)‡
		Wave 9	Jul 2014; Jan, May 2015		163,920 (23,232)‡
		Wave 10	Jul 2018; Jan, May 2019		137,471 (16,570)‡

Notes: ITC 4C = International Tobacco Control Four Country Survey (2002–2015). ITC 4CV = International Tobacco Control Four Country Smoking and Vaping Survey (2016–2020). BRFSS = US Behavioral Risk Factor Surveillance System. ACS = American Community Survey. TUS-CPS = Tobacco Use Supplement to the Current Population Survey. CATI = computer-assisted telephone interviewing. CAPI = computer-assisted personal interviewing. *66 observations had missing data for sex; 477,599 observations were used in the analysis. †1133 observations had missing data for sex; 429,836 observations were used in the analysis. ‡ Total sample size (sub-sample of adults who smoke) for TUS-CPS.

Supplementary Table 2. Baseline characteristics of ITC respondents at first wave of participation.

	Non-menthol			Menthol			Overall		
Characteristic	(n)	U %	W %	(n)	U %	W %	(n)	U %	W %
Census division*									
New England	(336)	4.1	5.1	(144)	3.7	4.5	(480)	4.0	4.9
Middle Atlantic	(995)	12.3	11.6	(634)	16.2	16.3	(1629)	13.6	13.1
East North Central	(1331)	16.4	15.4	(766)	19.6	17.5	(2097)	17.4	16.1
West North Central	(704)	8.7	7.8	(244)	6.2	5.9	(948)	7.9	7.2
South Atlantic	(1399)	17.3	17.4	(863)	22.1	22.6	(2262)	18.8	19.1
East South Central	(565)	7.0	7.1	(293)	7.5	7.5	(858)	7.1	7.2
West South Central	(855)	10.5	11.4	(383)	9.8	10.5	(1238)	10.3	11.1
Mountain	(635)	7.8	7.9	(187)	4.8	4.8	(822)	6.8	6.9
Pacific	(1289)	15.9	16.3	(397)	10.2	10.5	(1686)	14.0	14.4
First survey wave									
2002	(1525)	18.8	20.1	(577)	14.8	16.7	(2102)	17.5	19.1
2003	(483)	6.0	6.3	(193)	4.9	5.7	(676)	5.6	6.1
2004	(613)	7.6	8.0	(266)	6.8	7.8	(879)	7.3	8.0
2005	(538)	6.6	7.1	(192)	4.9	5.5	(730)	6.1	6.6
2006	(530)	6.5	6.9	(199)	5.1	6.1	(729)	6.1	6.6
2007	(506)	6.2	7.1	(192)	4.9	4.7	(698)	5.8	6.3
2008	(269)	3.3	3.5	(122)	3.1	3.5	(391)	3.3	3.5
2010	(237)	2.9	3.0	(130)	3.3	4.0	(367)	3.1	3.3
2013	(105)	1.3	1.5	(49)	1.3	1.1	(154)	1.3	1.4
2015	(1178)	14.5	15.7	(664)	17.0	18.6	(1842)	15.3	16.6
2016	(726)	9.0	5.6	(516)	13.2	6.3	(1242)	10.3	5.8
2018	(803)	9.9	9.1	(449)	11.5	10.2	(1252)	10.4	9.5
2020	(596)	7.3	6.0	(362)	9.3	9.8	(958)	8.0	7.2
Number of waves completed†									
One	(4580)	56.5	56.0	(2487)	63.6	62.8	(7067)	58.8	58.1
Two	(1687)	20.8	21.8	(727)	18.6	19.7	(2414)	20.1	21.1
Three or more	(1842)	22.7	22.2	(697)	17.8	17.5	(2539)	21.1	20.8
Sex									
Male	(3994)	49.3	55.6	(1656)	42.3	49.2	(5650)	47.0	53.6
Female	(4115)	50.7	44.4	(2255)	57.7	50.8	(6370)	53.0	46.4
Age group									
18–29	(1866)	23.0	22.4	(1267)	32.4	31.1	(3133)	26.1	25.2
30–49	(2835)	35.0	43.0	(1375)	35.2	42.2	(4210)	35.0	42.8
50+	(3408)	42.0	34.6	(1269)	32.4	26.6	(4677)	38.9	32.0
Race/ethnicity									
non-Hispanic White	(6708)	82.7	83.9	(2335)	59.7	58.2	(9043)	75.2	75.8
non-Hispanic Black	(276)	3.4	2.9	(921)	23.5	26.3	(1197)	10.0	10.3
Hispanic	(511)	6.3	5.7	(363)	9.3	8.4	(874)	7.3	6.6
non-Hispanic Other	(614)	7.6	7.5	(292)	7.5	7.1	(906)	7.5	7.3
Education									
≤ High school/not reported	(3437)	42.4	48.6	(1571)	40.2	48.0	(5008)	41.7	48.4
Some/completed college	(4672)	57.6	51.4	(2340)	59.8	52.0	(7012)	58.3	51.6
Income									
< $45,000/year	(4802)	59.2	60.4	(2412)	61.7	64.9	(7214)	60.0	61.9
≥ $45,000/yea	(3307)	40.8	39.6	(1499)	38.3	35.1	(4806)	40.0	38.1
Socioeconomic status									
Low	(2483)	30.6	34.8	(1189)	30.4	36.5	(3672)	30.5	35.3
Moderate	(3273)	40.4	39.4	(1605)	41.0	40.0	(4878)	40.6	39.6
High	(2353)	29.0	25.7	(1117)	28.6	23.6	(3470)	28.9	25.1
Smoking status									
Non-daily	(1024)	12.6	10.5	(556)	14.2	12.1	(1580)	13.1	11.0
Daily	(7085)	87.4	89.5	(3355)	85.8	87.9	(10440)	86.9	89.0
Cigarettes smoked/day									
≤ 10	(3126)	39.4	35.8	(1995)	52.3	48.8	(5121)	43.6	39.9
11–20	(3311)	41.8	43.8	(1378)	36.1	39.3	(4689)	39.9	42.3
21–30	(928)	11.7	12.4	(272)	7.1	7.6	(1200)	10.2	10.9
31+	(565)	7.1	8.0	(170)	4.5	4.3	(735)	6.3	6.8

Notes: U % = unweighted percentage; W % = weighted percentage. *US population distribution by census division in 2020: New England = 4.5%, Middle Atlantic = 12.8%, East North Central = 14.3%, West North Central = 6.5%, South Atlantic = 20.0%, East South Central = 5.9%, West South Central = 12.3%, Mountain 7.5%, Pacific = 16.2%. Source: Federal Reserve of Economic Data. (2023). Resident Population by Census Division, Annual. https://fred.stlouisfed.org/release/tables?rid=118&eid=259247&od=2020-01-01#. †Total number of ITC Survey waves completed.

Supplementary Table 3. Estimated regression coefficients for fixed effects and variance components from the final multilevel logistic regression model used to predict use of menthol cigarettes among adults who smoke in the US (n = 22,703 observations from 12,020 respondents participating in the International Tobacco Control US Surveys from 2002 to 2020.)

Parameter (reference)	Log OR	(Std Err)	t	df	p
Intercept	-1.307	(0.155)	-8.42	8	< 0.001
Sex (male)					
female	0.530	(0.145)	3.65	22,422	< 0.001
Age group (18-24)					
30–49	-0.393	(0.173)	-2.27	22,422	0.023
50+	-0.625	(0.124)	-5.02	22,422	< 0.001
Race/ethnicity (non-Hispanic White)					
non-Hispanic Black	2.586	(0.232)	11.17	24	< 0.001
Hispanic	0.927	(0.409)	2.26	24	0.033
non-Hispanic Other	0.236	(0.399)	0.59	24	0.561
Socioeconomic status (low)					
Moderate SES	0.067	(0.042)	1.57	22,422	0.116
High SES	0.131	(0.092)	1.43	22,422	0.153
Labor force participation rate*	-0.026	(0.014)	-1.92	22,422	0.055
Sex X race/ethnicity					
female X non-Hispanic Black	-0.412	(0.279)	-1.48	22,422	0.139
female X Hispanic	0.095	(0.365)	0.26	22,422	0.794
female X non-Hispanic Other	-0.184	(0.448)	-0.41	22,422	0.680
Age group X race/ethnicity					
30–49 X non-Hispanic Black	0.618	(0.305)	2.03	22,422	0.043
30–49 X Hispanic	-0.168	(0.395)	-0.42	22,422	0.671
30–49 X non-Hispanic Other	0.425	(0.406)	1.05	22,422	0.295
50+ X non-Hispanic Black	-0.271	(0.338)	-0.80	22,422	0.423
50+ X Hispanic	-0.146	(0.630)	-0.23	22,422	0.816
50+ X non-Hispanic Other	-0.111	(0.525)	-0.21	22,422	0.833
Sex X age group					
female X 30–49	-0.306	(0.163)	-1.87	22,422	0.061
female X 50+	-0.070	(0.132)	-0.53	22,422	0.594
Sex X age group X race/ethnicity					
female X 30–49 X non-Hispanic Black	0.350	(0.411)	0.85	22,422	0.395
female X 30–49 X Hispanic	0.501	(0.340)	1.47	22,422	0.140
female X 30–49 X non-Hispanic Other	-0.360	(0.457)	-0.79	22,422	0.431
female X 50+ X non-Hispanic Black	0.371	(0.392)	0.95	22,422	0.343
female X 50+ X Hispanic	-0.177	(0.443)	-0.40	22,422	0.690
female X 50+ X non-Hispanic Other	-0.071	(0.534)	-0.13	22,422	0.895
Piecewise linear trend†					
(1) Linear ("time")	0.008	(0.021)	0.40	22,422	0.688
(2) Lag, Camel Crush ("lagCC")	0.097	(0.024)	3.99	22,422	< 0.001
(3) First wave of ITC 4CV ("time4CV")	-0.117	(0.047)	-2.47	22,422	0.013
Covariance Parameters					
Census division (random intercept)	0.1569	(0.0573)			
Race/ethnicity (random intercept)‡	0.1015	(0.0393)			

Notes: See Supplementary Table 2, Model 8 for overall omnibus tests for fixed effects. Log OR = log odds ratio; Std Err = standard error; df = degrees of freedom. *Year-specific census division labor force participation rate, mean centred within divisions. †Piecewise linear trends were fit to model (1) a linear trend in the log odds of menthol use from 2002 to 2010, (2) a "bend" in the trend starting in 2010, allowing for market penetration of Camel Crush cigarettes into the US market following their introduction in 2008 (i.e., a lagged effect of Camel Crush cigarettes), and (3) a second bend in the trend to reflect the start of the ITC 4CV survey in 2016. ‡ The random intercept for race/ethnicity was nested within census divisions.

Supplementary Table 4. Agreement between modeled (ITC MrP) and direct (TUS-CPS) estimates of the census division prevalence of menthol use among adults who currently smoke in the US from 2002 to 2020 across estimated multilevel logistic regression models.

	Model 1			Model 2			Model 3		
Subgroup prevalence	oCCC	Precision	Accuracy	oCCC	Precision	Accuracy	oCCC	Precision	Accuracy
Overall	0.8973	0.9641	0.9307	0.8806	0.9583	0.9188	0.9165	0.9654	0.9494
Male	0.7785	0.9614	0.8098	0.7571	0.9478	0.7988	0.8080	0.9667	0.8358
Female	0.9366	0.9389	0.9975	0.9342	0.9403	0.9935	0.9350	0.9354	0.9995
Ages 18-29	0.7585	0.9289	0.8166	0.7668	0.9252	0.8287	0.7695	0.9230	0.8337
Ages 30-49	0.8420	0.9051	0.9304	0.8261	0.8987	0.9191	0.8705	0.9192	0.9470
Ages 50+	0.8721	0.8968	0.9725	0.8465	0.8835	0.9581	0.8730	0.8957	0.9747
non-Hispanic White	0.9185	0.9434	0.9737	0.8959	0.9341	0.9590	0.9317	0.9437	0.9873
non-Hispanic Black	0.2845	0.6843	0.4157	0.2972	0.7009	0.4240	0.2894	0.6777	0.4271
Hispanic	0.4578	0.6906	0.6629	0.4746	0.6873	0.6905	0.4675	0.6970	0.6707
All other groups	0.5789	0.5857	0.9883	**0.6111**	**0.6132**	**0.9967**	0.5685	0.5800	0.9801

	Model 4			Model 5			Model 6		
	oCCC	Precision	Accuracy	oCCC	Precision	Accuracy	oCCC	Precision	Accuracy
Overall	0.9147	0.9650	0.9478	0.8932	0.9632	0.9273	0.9275	0.9663	0.9599
Male	0.8055	0.9663	0.8336	0.7723	0.9589	0.8053	0.8226	0.9658	0.8517
Female	0.9347	0.9352	0.9995	0.9366	0.9396	0.9968	**0.9369**	**0.9377**	**0.9992**
Ages 18-29	0.7673	0.9230	0.8313	0.7584	0.9310	0.8146	0.7879	0.9291	0.8481
Ages 30-49	0.8681	0.9180	0.9457	0.8343	0.8997	0.9273	0.8760	0.9163	0.9559
Ages 50+	**0.8739**	**0.8963**	**0.9750**	0.8705	0.8962	0.9713	0.8698	0.8939	0.9730
non-Hispanic White	0.9306	0.9435	0.9863	0.9156	0.9432	0.9708	**0.9432**	**0.9493**	**0.9936**
non-Hispanic Black	0.2879	0.6770	0.4253	0.2850	0.6868	0.4150	0.2974	0.6844	0.4345
Hispanic	0.4659	0.6966	0.6689	0.4557	0.6870	0.6634	0.4723	0.6924	0.6822
All other groups	0.5655	0.5767	0.9806	0.5790	0.5851	0.9897	0.5588	0.5730	0.9752

	Model 7			Model 8			Model 9		
	oCCC	Precision	Accuracy	oCCC	Precision	Accuracy	oCCC	Precision	Accuracy
Overall	0.9295	0.9631	0.9651	**0.9315**	**0.9656**	**0.9647**	0.9097	0.9501	0.9575
Male	0.8287	0.9637	0.8599	**0.8330**	**0.9668**	**0.8616**	0.8192	0.9472	0.8648
Female	0.9316	0.9333	0.9982	0.9300	0.9314	0.9985	0.9191	0.9225	0.9963
Ages 18-29	0.7889	0.9256	0.8524	**0.8170**	**0.9249**	**0.8834**	0.8072	0.9174	0.8799
Ages 30-49	0.8792	0.9150	0.9609	**0.8846**	**0.9221**	**0.9594**	0.8242	0.8937	0.9223
Ages 50+	0.8683	0.8929	0.9724	0.8679	0.8985	0.9659	0.8700	0.8806	0.9880
non-Hispanic White	0.9425	0.9464	0.9959	0.9137	0.9311	0.9812	0.8844	0.9235	0.9577
non-Hispanic Black	0.2965	0.6776	0.4375	**0.5525**	**0.7488**	**0.7378**	0.4205	0.6939	0.6061
Hispanic	0.4713	0.6889	0.6842	**0.7001**	**0.8102**	**0.8641**	0.4659	0.6821	0.6831
All other groups	0.5381	0.5544	0.9706	0.5215	0.5318	0.9807	0.4937	0.5257	0.9392

Notes: oCCC = overall concordance correlation coefficient. Bolded values indicate the highest level of agreement between modeled and direct estimates of prevalence for a given subgroup and model.
